# Molecular dynamics simulation of the opposite-base preference and interactions in the active site of formamidopyrimidine-DNA glycosylase

**DOI:** 10.1186/s12900-017-0075-y

**Published:** 2017-05-08

**Authors:** Alexander V. Popov, Anton V. Endutkin, Yuri N. Vorobjev, Dmitry O. Zharkov

**Affiliations:** 1 SB RAS Institute of Chemical Biology and Fundamental Medicine, 8 Lavrentieva Ave., Novosibirsk, 630090 Russia; 2 Novosibrsk State University, 2 Pirogova St., Novosibirsk, 630090 Russia

**Keywords:** DNA glycosylase, 8-oxoguanine, Fpg, Molecular dynamics, Opposite-base specificity, Reaction mechanism

## Abstract

**Background:**

Formamidopyrimidine-DNA glycosylase (Fpg) removes abundant pre-mutagenic 8-oxoguanine (oxoG) bases from DNA through nucleophilic attack of its N-terminal proline at C1′ of the damaged nucleotide. Since oxoG efficiently pairs with both C and A, Fpg must excise oxoG from pairs with C but not with A, otherwise a mutation occurs. The crystal structures of several Fpg–DNA complexes have been solved, yet no structure with A opposite the lesion is available.

**Results:**

Here we use molecular dynamic simulation to model interactions in the pre-catalytic complex of *Lactococcus lactis* Fpg with DNA containing oxoG opposite C or A, the latter in either *syn* or *anti* conformation. The catalytic dyad, Pro1–Glu2, was modeled in all four possible protonation states. Only one transition was observed in the experimental reaction rate pH dependence plots, and Glu2 kept the same set of interactions regardless of its protonation state, suggesting that it does not limit the reaction rate. The adenine base opposite oxoG was highly distorting for the adjacent nucleotides: in the more stable *syn* models it formed non-canonical bonds with out-of-register nucleotides in both the damaged and the complementary strand, whereas in the *anti* models the adenine either formed non-canonical bonds or was expelled into the major groove. The side chains of Arg109 and Phe111 that Fpg inserts into DNA to maintain its kinked conformation tended to withdraw from their positions if A was opposite to the lesion. The region showing the largest differences in the dynamics between oxoG:C and oxoG:A substrates was unexpectedly remote from the active site, located near the linker joining the two domains of Fpg. This region was also highly conserved among 124 analyzed Fpg sequences. Three sites trapping water molecules through multiple bonds were identified on the protein–DNA interface, apparently helping to maintain enzyme-induced DNA distortion and participating in oxoG recognition.

**Conclusion:**

Overall, the discrimination against A opposite to the lesion seems to be due to incorrect DNA distortion around the lesion-containing base pair and, possibly, to gross movement of protein domains connected by the linker.

**Electronic supplementary material:**

The online version of this article (doi:10.1186/s12900-017-0075-y) contains supplementary material, which is available to authorized users.

## Background

Formamidopyrimidine-DNA glycosylase (Fpg or MutM) is a bacterial DNA repair enzyme that removes several abundant oxidized bases from DNA. The principal substrate bases of Fpg are 8-oxoguanine (oxoG), 2,6-diamino-4-oxo-5-formamidopyrimidine (fapyG) and 2,4-diamino-5-formamidopyrimidine (fapyA) [1, 2] but the enzyme also can recognize several dozens of other damaged purines and pyrimidines [3–10]. By excision of a damaged base, Fpg initiates base excision repair (BER), which engages AP endonucleases, a DNA polymerase and a DNA ligase to restore the integrity of DNA [11, 12].

The activity of Fpg towards oxoG has attracted much attention due to abundance and biological importance of this lesion, induced in DNA by oxidative metabolism byproducts, oxidative stress, and ionizing radiation [13]. Steric and electrostatic repulsion between the substituent at C8 and the sugar–phosphate atoms effectively pushes oxoG towards the *syn* conformation, in which oxoG forms a Hoogsteen pair with A [14, 15]. Misincorporation of A by DNA polymerases, in the absence of repair, leads to a G → T transversion after the second round of replication.

Systems for repair of oxoG have been found in all cellular organisms. The tendency of oxoG to form pairs with both C and A presents a challenge to its repair: both oxoG:C and oxoG:A pairs must be converted into G:C pairs. This requirement is not trivial since a simple removal of oxoG from an oxoG:A mispair would generate a G → T transversion after the repair. This problem is circumvented by repair of oxoG:A pairs in two sequential rounds of BER [16]. The non-damaged (but inappropriately incorporated) A is removed first and replaced with C, and the resulting oxoG:C pair is then repaired through the excision of oxoG. In *E. coli*, the mutagenic potential of oxoG is counteracted by three enzymes, Fpg, MutT, and MutY, collectively known as a “GO system”. Fpg excises oxoG from oxoG:C pairs but has little activity towards oxoG:A substrates to prevent G → T transversions [1, 17]. Another DNA glycosylase, MutY, specifically excises A from A:oxoG mispairs. If G in DNA is oxidized to oxoG, it will inevitably be paired with C and will be removed by Fpg. If, on the other hand, A is incorporated during replication opposite an unrepaired oxoG, the resulting oxoG:A mispair will be a substrate for MutY but not for Fpg. The repair synthesis then has a chance to incorporate C opposite oxoG [16].

**Fig. 1:**
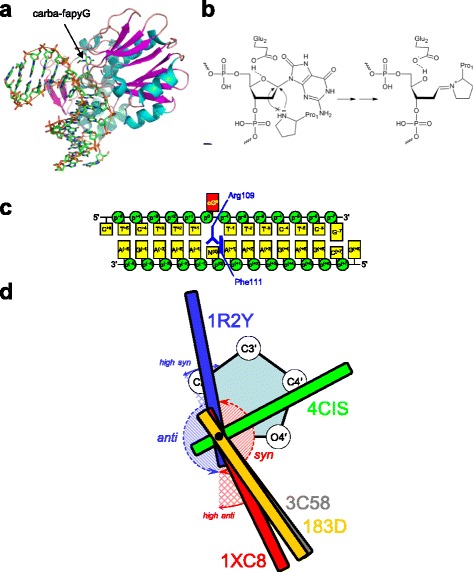
**a,** Structure of *Lla*-Fpg (1XC8) used as a starting model. The protein is colored according to its secondary structure (*cyan*, α helices; *magenta*, β sheets; *coral*, loops); the DNA is colored by atom type (*green*, C; *blue*, N; *red*, O; *orange*, P). An *orange* line is drawn through P atoms in DNA to highlight an axial kink induced by Fpg binding. **b,** Mechanism of oxoG excision by Fpg proposed from the structural data [35]. The S_N_2 displacement occurs in the C1′ → O4′ direction rather than in the C1′ → N9 direction. **c,** Schematic representation of the modeled DNA duplex and numbering of DNA bases and phosphates (p). N^(0)^ is either C or A. Positions of Arg109 and Phe111 in the complex are indicated. **d,** Schematic position of the damaged base relative to the sugar plane in the structures of free oxoG-containing DNA (183D, [56]) or Fpg–DNA complexes containing various purine-derived lesions everted into the active site (1XC8, 3C58, 4CIS and 1R2Y; see structure details in the text)

The function of the GO system therefore critically depends on the selectivity of Fpg to the base opposite to the damaged one. X-ray structures are available for free Fpg protein from *Thermus thermophilus* (*Tth*-Fpg) and for various types of complexes of DNA with Fpg from *Escherichia coli* (*Eco*-Fpg), *Geobacillus stearothermophilus* (*Bst*-Fpg) and *Lactococcus lactis* (*Lla*-Fpg) [18–33] (Fig. 1a). Based on these structures, kinetic data, and computational modeling, a reaction mechanism has been suggested that involves a nucleophilic attack at C1′ of oxoG by a lone electron pair of the secondary amino group of the deprotonated N-terminal Pro1 residue, assisted by protonation of O4′ in the deoxyribose moiety [33–36]. As a result, the *N*-glycosidic bond is broken, the deoxyribose ring is opened, and a Schiff base covalent intermediate between Fpg and DNA is formed (Fig. 1b). This series of events is followed by two sequential steps of elimination of the 3′- and 5′-phosphates and hydrolysis of the Schiff base. However, many questions about the initial stages of the reaction still remain. For example, the mechanism of oxoG recognition in the active site of the enzyme is unclear, and the mechanism of proton transfer in the multistep reaction is unknown. Notably, no structural or modeling data is available for Fpg in a complex with oxoG:A-containing DNA, limiting our knowledge of the mechanisms of rejection of this functionally relevant mispair. In this work, we use molecular dynamics approach to analyze the structure of complexes of Fpg with oxoG-containing DNA (either A or C opposite the lesion) to get an insight into the reasons behind the opposite-base selectivity of the enzyme and into the dynamic features of the immediate pre-catalytic complex involving oxoG.

## Methods

### Model preparation

The starting model for the MD analysis of Fpg bound to oxoG-containing DNA was the X-ray structure of *Lla*-Fpg in a complex with a 14-mer DNA duplex (Fig. 1a,c) containing a non-hydrolysable carbocyclic analog of fapyG (PDB ID 1XC8) [23]. The lesion was changed to oxoG using the following protocol. The initial structure of the oxoG base was taken from *Bst*-Fpg coordinates (PDB ID 1R2Y) [22]. The base was aligned for the best fit to the fapyG ring and incorporated into the PDB file instead of fapyG. The methylene group in the cyclopentane ring isosteric to O4′ was manually changed to oxygen. Heavy atoms of the side chains lacking in the structure were built using the Missing Heavy Atom Restoration module of BioPASED molecular modeling package [37]. Out of 397 water molecules found in the crystal unit cell, seven that reside in the enzyme’s active site or in its immediate vicinity were retained as explicit water, otherwise the modeling was done in implicit water to broaden the sampled conformational space. To analyze the effect of A *vs* C placed opposite oxoG, three sets of simulations were performed: one with oxoG opposite C (henceforth termed C models), another with oxoG opposite A(*anti*) (Aa models), and the third, with oxoG opposite A(*syn*) (As models). All models containing A opposite to the lesion were constructed by base replacement. For each group, four simulations were done, with different protonation states of Pro1 and Glu2: deprotonated Pro1 and Glu2 (PRN-GLU models), protonated Pro1 and deprotonated Glu2 (PRO-GLU), deprotonated Pro1 and protonated Glu2 (PRN-GLH), and protonated Pro1 and Glu2 (PRO-GLH models) (Table 1). The starting structures were checked for errors using the PDB Validator tool of BioPASED [37]. All models were energy-minimized in 500 steps of Fletcher energy optimization algorithm and finally refined by simulated annealing MD for 500 ps using the BioPASED package [37]. The AMBER force field parameters for oxoG and neutral Pro1 were from [38]. Force field parameters for neutral glutamate were from AMBER ff99 [39]. The parameters for the rest of the protein, including Zn^2+^, were taken from the classic Amber ff99 force field. The protonation states of the other residues were selected to match physiological pH conditions; e. g., E5 was modeled negatively charged. The Zn^2+^ ion was described as a single atom with four distance-based harmonic restraints to bind it to the coordinating cysteins and to maintain the correct geometry. Implicit counterion correction was applied by scaling charges of phosphate groups by a factor of 0.2 [40].

**Table 1:** Mean r.m.s.d. values of the models and their standard deviations (Å) over the last 8 ns of the runs

	PRN-GLH	PRN-GLU	PRO-GLH	PRO-GLU
	Global			
oxoG:C	1.50 ± 0.06	1.58 ± 0.07	1.69 ± 0.07	1.51 ± 0.05
oxoG:A(*anti*)	1.75 ± 0.05	1.43 ± 0.06	1.66 ± 0.06	1.45 ± 0.06
oxoG:A(*syn*)	1.68 ± 0.06	1.53 ± 0.06	1.41 ± 0.06	1.74 ± 0.06
	Active site			
oxoG:C	1.37 ± 0.10	1.55 ± 0.08	1.60 ± 0.13	1.58 ± 0.11
oxoG:A(*anti*)	1.89 ± 0.06	1.57 ± 0.08	2.16 ± 0.06	1.86 ± 0.07
oxoG:A(*syn*)	1.66 ± 0.12	1.61 ± 0.06	1.44 ± 0.10	1.92 ± 0.13

### Molecular dynamics

Molecular dynamics simulations (10 ns) were performed using the BioPASED molecular dynamics modeling software [37] using the AMBER ff99 force field with BioPASED modifications and EEF1 analytical implicit solvent model [41], with an integration time step of 1 fs. The system was gradually heated from 10 K to 300 K during 50 ps and equilibrated at this temperature (the heating time was 150, 200, and 250 ps in the repeat runs of the PRO-GLH models). A classic molecular dynamics trajectory was generated in the NVT ensemble with harmonic restraints of 0.001 kcal/A^2^ for the protein and water atoms, 0.25 kcal/A^2^ for the atoms of the terminal nucleotides, and 0.0025 kcal/A^2^ for the rest of the DNA atoms. Coordinates of each atom of the system were saved each 2 ps, thus producing a trajectory size of 5000 snapshots. The trajectories were analyzed using MDTRA [42], a part of the BISON package [43]. Trajectories were compared using moving MWZ method [44] with bins of 50 snapshots. Statistically significant differences in parameters between different models were estimated using F-test, with false discovery rate method (Benjamini–Hochberg procedure) employed to correct for multiple comparisons [45]. Hydrogen bonds were searched using MDTRA [42]. Structures were visualized and rendered using VMD [46], RasMol [47] and PyMOL (Schrödinger, Portland, OR).

### pH dependence of Fpg activity

*Eco*-Fpg was purified as described [19]. Oligonucleotides were synthesized in-house from commercially available phosphoramidites (Glen Research, Sterling, VA). An oligonucleotide 5′-CTCTCCCTTCXCTCCTTTCCTCT-3′ (X = oxoG) was ^32^P-labeled using polynucleotide kinase (SibEnzyme, Novosibirsk, Russia) and γ[^32^P]ATP (PerkinElmer, Waltham, MA) following the manufacturer’s protocol and annealed to a complementary strand placing C or A opposite oxoG. The reactions (20 μl) included 25 mM sodium phosphate buffer (H_3_PO_4_/NaH_2_PO_4_, NaH_2_PO_4_/Na_2_HPO_4_, and Na_2_HPO_4_/Na_3_PO_4_ conjugate pairs spanning the range of pH 4.0–9.0), 100 nM duplex oligonucleotide substrate, and either 2 nM (steady-state experiments) or 500 nM Fpg (single-turnover experiments). The reaction was allowed to proceed for 1 min either at 37 °C with 2 nM Fpg or at 0 °C with 500 nM Fpg, and was stopped by adding 20 μl of formamide/EDTA gel loading dye and heating at 95° for 3 min. The products were separated by electrophoresis in 20% polyacrylamide gel/8 M urea and quantified by phosphorimaging using a Molecular Imager FX system (Bio-Rad Laboratories, Hercules, CA). Three independent experiments have been performed. Calculation of p*K*_a_ for Pro1 and Glu2 were done using PROPKA v3.1 [48]. Circular dichroism spectra were recorded on a JASCO J-600 CD spectrometer (JASCO Analytical Instruments, Tokyo, Japan) at 25 °C in 30 mM Na phosphate with a 1-nm step.

### Evolutionary analysis

A taxonomically balanced sample of 124 bacterial Fpg sequences (limited to two sequences per taxonomic family) was constructed by protein BLAST search [49] in the NCBI non-redundant protein sequences database using *Eco*-Fpg as a query, filtered for conservation of the N-terminal Pro–Glu dipeptide and C-terminal zinc finger, and clipped from the absolutely conserved N-terminal Pro to the absolutely conserved Gln after the fourth Cys of the zinc finger as described [50, 51]. Alignment of multiple sequences and neighbor-joining tree construction was performed using Clustal Omega [52]. Hierarchical analysis of conservation of physicochemical properties in the alignments was done using AMAS [53] with 5% atypical residues allowed; the results are reported as conservation numbers (*C*_*n*_).

## Results and discussion

### General model considerations

#### Selection of the starting structure

Currently, the Protein Data Bank holds 56 released X-ray structures of Fpg, belonging to four bacterial species and sampling several points along the reaction coordinate [18–33]. Our selection of the starting structure for MD was guided by the following considerations. First, it should contain DNA with the damaged base still in place, residing in the enzyme’s active site. Second, minimal deviation from the wild-type enzyme recognizing oxoG should be present. Third, the structure should have good resolution (<2.0 Å), with as few as possible residues missing.

Based on these considerations, we have chosen 1XC8, the 1.95-Å structure of wild-type *Lla*-Fpg bound to DNA containing a non-cleavable carbacyclic fapyG analog (carba-fapyG [23]) as a starting model (Fig. 1a). In carba-fapyG, a methylene group substitutes for O4′, and the damaged base, fapyG, is different from oxoG only by the absence of a bond between N9 and C8 of the purine heterocycle O4′ [54]. The *Lla*-Fpg is nearly identical to *Eco*-Fpg in its selectivity for C *vs* A as the opposite base [17].

#### OxoG glycosidic angle

Besides 1XC8, the structures of Fpg bound to DNA with an extrahelical damaged base include *Lla*-Fpg bound to DNA containing carbacyclic *N*5-benzyl-fapyG (3C58 [26]), carbacyclic oxoG (4CIS [33]) or 5-hydroxy-5-methylhydantoin (2XZF, 2XZU [29]) and *Bst*-Fpg bound to DNA containing oxoG (1R2Y) or 5,6-dihydrouracil (1R2Z) [22]. With the exception of 1R2Y and 4CIS, the damaged bases in the structures are quite different from oxoG. In the 1R2Y structure, oxoG is present in DNA, and the cleavage is prevented by changing the absolutely conserved catalytic Glu2 residue into Gln [22]. In this structure, oxoG is often stated to be in the *syn* conformation in the active site, yet its χ angle (101°) is actually in the *anti* domain (namely, in its border range, so-called “*high syn*”) (Fig. 1d). On the contrary, oxoG opposite C in B-DNA is usually stated to be *anti* as it forms regular Watson–Crick bonds [55, 56], yet its χ angle in the crystal structure (–55°, [56]) is actually in the *syn* domain. Carba-fapyG in 1XC8 is in the *high anti* range (χ = –64°), and only in 4CIS, carba-oxoG is unambiguously *syn* (χ = 27°, Fig. 1d). Moreover, oxoG in 4CIS does not form the same set of hydrogen bonds with the active site as in 1R2Y. The possibility of conformation artifacts induced by E2Q mutation has been amply discussed in the literature [23, 38, 57–61]. Therefore, we have chosen 1XC8, which straddles the *syn*/*anti* border (Fig. 1d) as our starting model and allowed the conformation to drift into the most preferable χ range during MD.

#### Opposite base glycosidic angle

While the oxoG:A pair in B-DNA exists as oxoG(*syn*):A(*anti*), this does not mean that the same conformations will be observed in the complex with Fpg, since the hydrogen bonds within the mispair are lost upon oxoG eversion, and the conformation of the nucleotides is governed largely by their interaction with the protein residues. The most relevant example is given by another oxoG mispair, oxoG:G, which adopts the conformation oxoG(*syn*):G(*anti*) in the B-DNA duplex [62] but the G opposite to the lesion flips into *syn* and forms two strong hydrogen bond with an Arg residue when this duplex is bound to *Bst*-Fpg [20]. Therefore, in addition to the oxoG:C pair, we have constructed two series of models with oxoG:A, with A in either *anti* or *syn* conformation to fully explore the range of possible dynamics of Fpg–oxoG:A complex.

#### Solvent

Although MD in explicit solvent is common nowadays, recent advances in implicit solvent models revived the popularity of this alternative [63–65]. The major advantages of implicit solvent over the explicit one are speed, better estimates of solvation and folding energy, wider coverage of conformational space and more accurate account for pH and residue ionization. The latest versions of Poisson–Boltzmann, generalized Born and hybrid implicit/explicit models are comparable with explicit solvent-based calculations with respect to agreement with experimental free energy data [63–65]. Although the acceleration of conformation sampling afforded by implicit solvent strongly depends on the modeled system, direct comparisons show a 7–10-fold increase for a system with several conformational transitions [66]. Since our primary interest was to sample a wide range of conformations available for the Fpg–substrate complexes, we have chosen a hybrid model combining an implicit solvent with explicit strongly bound water molecules; such approaches retain the advantages of implicit methods but significantly improve quality of protein–DNA interface models [67].

#### Protonation state of the catalytic dyad

The ionizable groups of Pro1 and Glu2 directly participate in the enzymatic reaction. Mechanistically, the nucleophilic attack by Pro1 at C1′[oxoG] requires N[Pro1] to carry a lone electron pair (Fig. 1b). On the other hand, opening of the deoxyribose ring involves protonation of its O4′, which is near Oε2 of Glu2 (Fig. 1b); quantum mechanics/molecular mechanics (QM/MM) simulations show that O4′ protonation provides a low-barrier path to glycosidic bond cleavage by Fpg and its eukaryotic functional analog, OGG1 [33, 36, 68]. From several structures Fpg–DNA complexes, it has been suggested that the proton is shuttled from N[Pro1] to Oε2[Glu2], perhaps through a network of crystallographic water molecules present in the active site [19, 26]; this possibility was also favored by QM/MM analysis [69]. However, no attempt to estimate p*K*_a_ of Pro1 and Glu2 has been reported in the literature. It is possible that a mixture of Pro1/Glu2 ionization states exists in the active centers of different Fpg molecules at physiological pH; although only one of them (PRN-GLH) is permissive for the reaction chemistry, the path to this state may go through other states. Therefore, we have performed MD of the full system for four ionization states of the Pro1–Glu2 catalytic dyad: PRN-GLH, PRN-GLU, PRO-GLH, and PRO-GLU (see Methods).

#### Overall model characterization

The root mean square deviation (r.m.s.d.) with respect to the backbone of the starting structure of the complex was calculated every 2 ps. R.m.s.d. values of all the models increased rapidly during the first 500 ps of the dynamics and stabilized at approximately 1.6 Å (see Table 1 and Additional file 1: Fig. S1). Overall r.m.s.d. of all models was similar; however, in the active center (defined as all protein residues with at least one atom within 4 Å of oxoG nucleotide or the opposite C/A nucleotide, plus three nucleotide pairs centered on the oxoG), the r.m.s.d. of the C models was significantly lower than in the A models (*p* < 10^–4^). The DNA backbone displayed higher mobility than the protein backbone: average r.m.s.d. of the DNA residues was greater by 0.54–0.98 Å depending on the model. The overall complex conformation was stable along the whole trajectory with a radius of gyration ~20 Å for each model (20.17 ± 0.04 to 20.30 ± 0.05 Å). The angle of DNA kink was also stable (55° ± 2° to 63° ± 2°, depending on the model).

#### Simulation reproducibility

To test the consistence of results between independent runs, we have selected three models (PRO-GLH-C, PRO-GLH-Aa, and PRO-GLH-As) and performed three additional simulations with each one, to the total of nine additional simulations, using different heating times (150, 200, and 250 ps) to provide different conditions for the start of the production run. Then the resulting four trajectories for each model (one original and three new) were compared. The r.m.s.d. values of individual runs were similar (1.0–1.5 Å over the last 8 ns, Additional file 1: Fig. S1). The inter-run r.m.s.d. were expectedly higher (1.9–2.2 Å, Additional file 1: Fig. S1) but still did not show significant divergence of the models. Stable hydrogen bonds, including model-specific ones, were well consistent across the four runs (Additional file 1: Fig. S5B); the 90% cut-off of the mean occurrence identified as stable all Watson–Crick bonds and 79% of the main-chain bonds observed in the 1XC8 crystal structure.

### Pro–Glu catalytic dyad

#### Arrangement of the reacting groups in the models

**Fig. 2:**
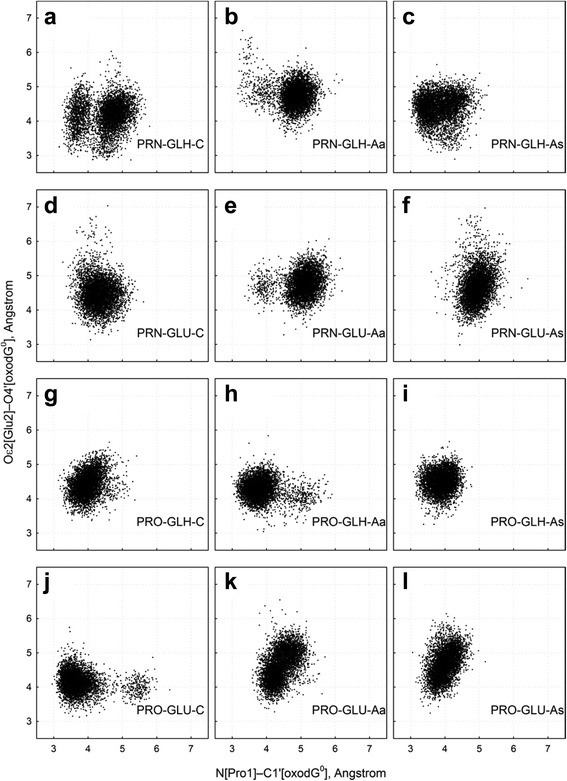
Distances N[Pro1]…C1′[oxoG^0^] and Oε2[Glu2]…O4′[oxoG^0^] during the simulation with different protonation states of N[Pro1] and Oε2[Glu2] (**a–l,** the model nature is indicated in the respective panels)

We have sampled the population of two key distances of the Fpg–DNA complex, N[Pro1]…C1′[oxoG] and Oε2[Glu2]…O4′[oxoG] in all our models (Fig. 2, Table 2). In all C models (Fig. 2a, D, G, J), the distribution of Oε2[Glu2]…O4′[oxoG] distances was unimodal and produced similar central values (Table 2). On the contrary, the N[Pro1]…C1′[oxoG] distance was less stable: in some models, two peaks in the distribution histogram were clearly observed, indicative of stable conformational basins (Table 2).

**Table 2:** Key distances and angles around the reacting C1′ atom of oxoG

Model		Distance N[Pro1]… C1′[oxoG^0^], Å	Distance Oε2[Glu2]… O4′[oxoG^0^], Å	Angle C1′[oxoG^0^]… N[Pro1]… Cδ[Pro1], degrees	Angle O4′[oxoG^0^]… C1′[oxoG^0^]…N[Pro1], degrees	# of snapshots with optimal geometry
C	PRN-GLH	3.73 (3.41–4.04)^*a*^ 4.74 (4.29–5.20)	4.20 (3.41–4.79)	61 (55–95)	150 (125–169)	409
	PRN-GLU	4.31 (3.83–4.88)	4.46 (3.87–5.12)	116 (80–122)	168 (145–171)	293
	PRO-GLH	3.97 (3.55–4.48)	4.42 (3.90–4.96)	119 (113–122)	165 (156–168)	1077 (788)^*b*^
	PRO-GLU	3.59 (3.27–4.17) 5.43 (5.01–5.84)	4.13 (3.68–4.60)	107 (78–122)	145 (135–151)	141
A (*anti*)	PRN-GLH	4.86 (4.06–5.30)	4.75 (4.24–5.27)	52 (49–77)	153 (136–157)	0
	PRN-GLU	5.12 (4.35–5.57)	4.76 (4.22–5.29)	52 (49–75)	158 (132–165)	0
	PRO-GLH	3.79 (3.34–4.78)	4.27 (3.83–4.74)	100 (89–107)	141 (136–152)	73 (277)
	PRO-GLU	4.39 (3.96–4.97)	4.62 (3.92–5.33)	124 (94–133)	168 (156–172)	78
A (*syn*)	PRN-GLH	3.82 (3.26–4.54)	4.44 (3.71–4.94)	101 (89–113)	155 (136–167)	225
	PRN-GLU	4.94 (4.52–5.38)	4.65 (4.01–5.48)	55 (48–62)	149 (135–160)	0
	PRO-GLH	3.92 (3.43–4.31)	4.49 (4.02–4.95)	119 (107–122)	167 (157–170)	961 (137)
	PRO-GLU	3.99 (3.57–4.43)	4.65 (4.02–5.34)	118 (93–121)	154 (144–164)	184

^*a*^Median and 90% range in parentheses

^*b*^Average over three repeat runs in parentheses

**Fig. 3:**
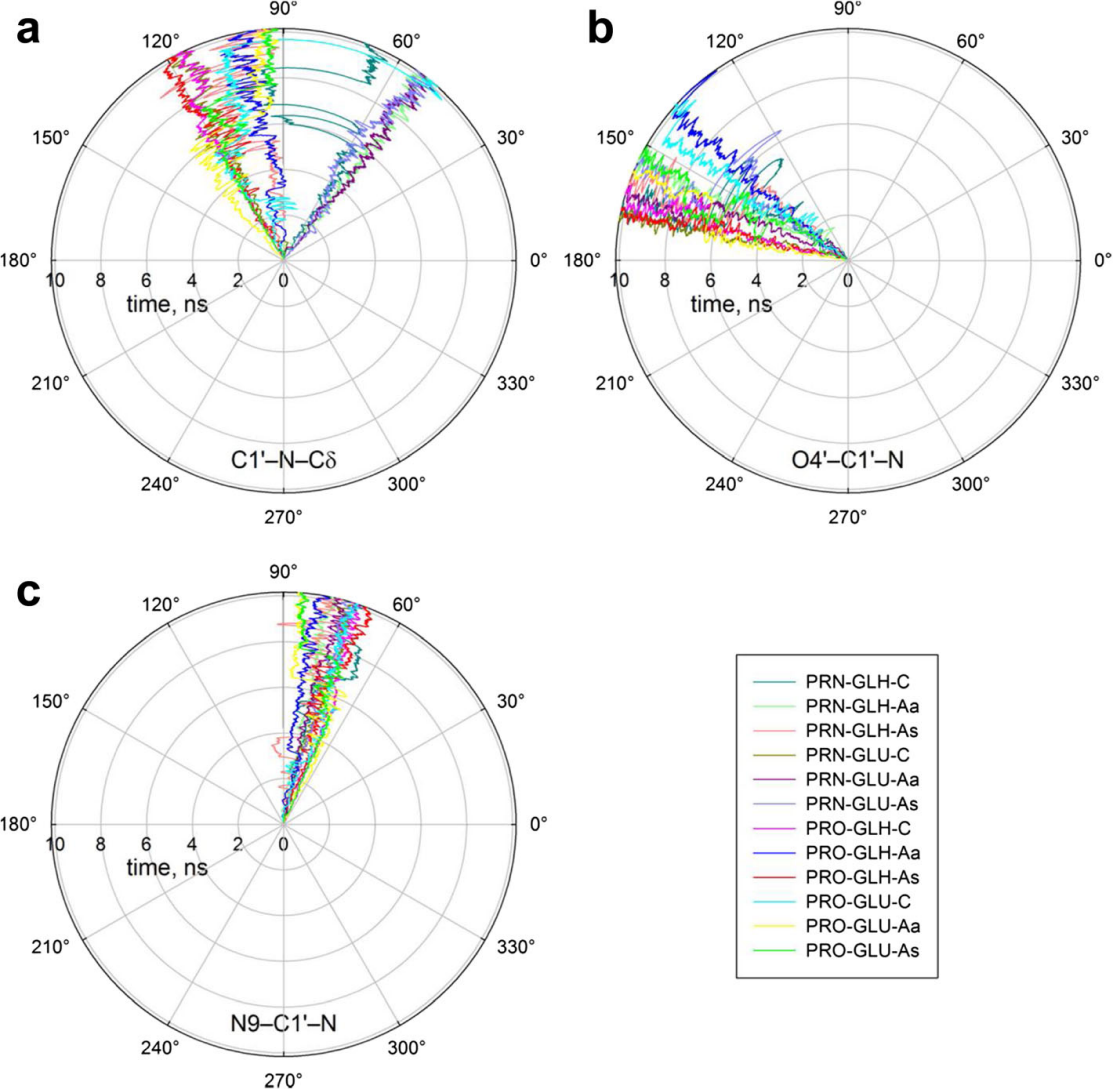
Angles C1′[oxoG^0^]…N[Pro1]…Cδ[Pro1] **(a)**, O4′[oxoG^0^]…C1′[oxoG^0^]…N[Pro1] **(b)** and N9[oxoG^0^]…C1′[oxoG^0^]…N[Pro1] **(c)** in the models. Moving average of 50 consecutive snapshots is plotted vs time. The traces are color-coded: *dark cyan*, PRN-GLH-C; *light lime*, PRN-GLH-Aa; *coral*, PRN-GLH-As; *olive*, PRN-GLU-C; *dark magenta*, PRN-GLU-Aa; *light blue*, PRN-GLU-As; *magenta*, PRO-GLH-C; *blue*, PRO-GLH-Aa; *red*, PRO-GLH-As; *cyan*, PRO-GLU-C; *yellow*, PRO-GLU-Aa; *green*, PRO-GLU-As

Another important parameter in the reaction of base excision is the angle of attack by Pro1 at C1′. Two mechanisms for S_N_2 displacement initiating Schiff base formation have been considered for bifunctional DNA glycosylases: with the C1′–O4′ bond or C1′–N9 bond breaking first [35, 70]. Enzyme-catalyzed S_N_2 reactions require a 10°–20° alignment of the nucleophile lone pair and carbon antibonding orbital [71, 72]. The ideal attack geometry for Pro1 is thus 107° for the C1′[oxoG]…N[Pro1]…Cδ[Pro1] angle and 180° for the X…C1′[oxoG]…N[Pro1] angle where X is either O4′[oxoG] or N9[oxoG]. As can be seen from Fig. 3 and Table 2, the C1′…N…Cδ angle of all PRO models, as well as PRN-GLH-C, PRN-GLH-As, and PRN-GLU-C, either lied in the acceptable domain or made appreciable excursions to it. All models were incompatible with the C1′–N9 direction of nucleophilic attack (Table 2). The O4′…C1′…N angle for 7 of 12 models lied in the acceptable domain, and was close to this range in other models, consistent with the C1′–O4′ attack. The opposite base had no consistent effect on the Pro1 approach angle.

Defining the “optimal geometry” as N[Pro1]…C1′[oxoG] distance < 4 Å, Oε2[Glu2]…O4′[oxoG^0^] distance < 4.5 Å, and C1′[oxoG^0^]…N[Pro1]…Cδ[Pro1] and O4′[oxoG]…C1′[oxoG]…N[Pro1] within 20° of the ideal values, we have sampled the population of the zone with all four parameters optimal (Table 2). All A(*anti*) models showed the optimal geometry very rarely. For C and A(*syn*) models, PRO-GLH was most populated, followed by PRN-GLH. Interestingly, PRN models were more selective towards C vs A(*syn*). Other sensible definitions of “optimal” Pro1 and Glu2 distances (e. g., the lowest quartile of the respective distance population), also showed C models spending more time in the optimal conformation than A models. The preference of C models for the optimal geometry was also evident in the repeat runs of the PRO-GLH models (Table 2).

#### *p*K_a_ estimate of the catalytic dyad residues

To get an independent estimate of the protonation state of Pro1 and Glu2, we have used PROPKA, an empirical algorithm based on the spatial proximity of charged residues [48]. In addition to our starting structure, we have considered several other PDB structures of Fpg from different species (Additional file 2: Table S1). In all cases, p*K*_a_ of Pro1 was notably lowered (by 0.35–2.99 units) compared with the reference p*K*_a_ of N-terminal Pro, while p*K*_a_ of Glu2 was considerably higher (by 1.54–3.64 units) than the reference p*K*_a_ of the internal Glu side chain. Similar p*K*_a_ changes were reported for phage T4 endonuclease V, another DNA glycosylase employing the N-terminal amino group and a Glu carboxyl as a catalytic dyad [73]. Interestingly, structures of free Fpg and Fpg bound to undamaged DNA with the sampled base still intrahelical displayed more acidic p*K*_a_ for Glu2, suggesting that this group may be specifically activated upon eversion of the damaged nucleotide. Although PROPKA considers the influence of nucleic acid ligands on amino acid ionization potential only approximately, it is nevertheless clear that in Fpg, Pro1 is considerably more acidic, and Glu2, more basic than expected.

#### pH profile of Fpg activity

**Fig. 4:**
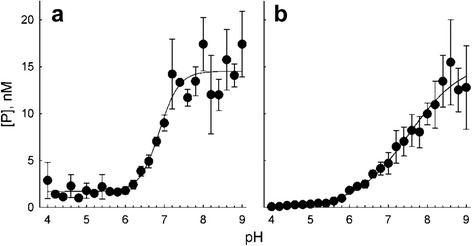
pH dependence of Fpg activity. **a,** single-turnover conditions (500 nM Fpg, 100 nM substrate, 0 °C). **b,** steady-state conditions (2 nM Fpg, 100 nM substrate, 37 °C). See Methods for details

To assess the functional importance of the catalytic dyad protonation states experimentally, we have analyzed the pH profile of activity for *Eco*-Fpg, assuming that the mechanistic features of base excision will be conserved in *Eco*-Fpg and *Lla*-Fpg. Usually, when an enzyme’s active site possesses two functionally important ionizable groups, one of which has to be protonated while the other has to be deprotonated for activity, the pH dependence is characteristically bell-shaped. For DNA glycosylases, such bell-shaped dependence was shown for human alkyladenine glycosylase, which is monofunctional, structurally different from Fpg, and uses a histidine and a glutamate residue as a general acid and a general base, respectively [74]. On the contrary, Fpg showed a single transition in the activity over a pH range of 5 units (pH 4 to pH 9) (Fig. 4). This was observed both under single-turnover conditions, where the rate is limited by the catalytic step of the reaction (Fig. 4a) and steady-state conditions, where the reaction rate, in the case of Fpg, is a function of both the catalytic step and product release (Fig. 4b). The p*K*_a_ values calculated from a two-state model were 6.8 ± 0.1 and 7.5 ± 0.3 for the single-turnover and steady-state conditions, respectively; the increase in p*K*_a_ under the steady-state conditions is likely due to a pH effect on the partially rate-limiting product release step. Circular dichroism spectra showed no considerable change in the Fpg structure at pH 4 (Additional file 1: Fig. S2), so the activity changes most likely can be assigned to the ionization of critical active site residues.

Since Pro1 has to be deprotonated for the reaction, the rising activity *vs* pH plot with a single inflection may be explained by this deprotonation and suggests that the equilibrium ionization state of Glu2 is not rate-limiting. As discussed below, Glu2 may be conveniently protonated by a water molecule trapped in the active site.

#### Other interactions of the catalytic dyad

Pro1 did not form stable interactions within the active site in the models, consistent with the available structural information on the pre-catalytic Fpg complexes. In contrast, in 1XC8 and other known structures of Fpg, Glu2 accepts two hydrogen bonds from the amides of Ile172 and Tyr173 (or their counterparts in other Fpgs). These bonds were stable in all our simulations independently of the protonation state of Glu2. Several available structures (1K82, 1L1T, 1L1Z [19, 20]) strongly suggest that these two bonds hold the carboxylic group of Glu2 in a position suitable for interaction with a nearby water molecule, and, later in the reaction, with O4′ of the damaged nucleotide that becomes a hydroxyl after base excision and sugar ring opening. Importantly, a water molecule (see the section “Water molecules in the Fpg–DNA complex”) was the only stably interacting group other than Ile172 and Tyr173, and this interaction was not affected by the protonation state of Glu2.

### Fpg interactions with oxoG and the opposite base

#### Stability of oxoG in the base-binding pocket

**Fig. 5:**
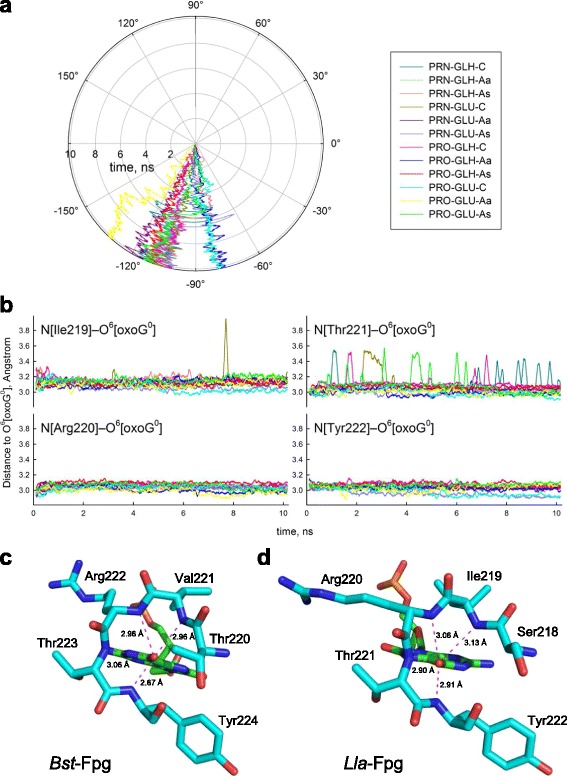
**a,** χ angle evolution during the simulation. **b,** distances between O^6^[oxoG^0^] and main chain amide nitrogen atoms of Ile119, Arg220, Thr221, and Tyr222. Moving average of 50 consecutive snapshots is plotted *vs* time. The colors of the traces are the same as in Fig. 3. **c,** loop Thr220–Tyr224 of *Bst*-Fpg forms an extensive set of contacts with O^6^ of oxoG in *high syn* orientation (χ = 101°, 1R2Y). **d,** a homologous loop Ser218–Tyr222 of *Lla*-Fpg forms the same set of contacts with O^6^ when oxoG is flipped around the glycosidic bond (χ = –103°, PRO-GLH-C model, 9 ns)

To inquire what features of Fpg–substrate interactions may explain poor substrate properties of oxoG:A mispairs, we have compared the dynamics of C, Aa and As models. The eversion angle of oxoG [75] was similar in all models (median range 74°–87°), indicative of full insertion of the damaged nucleoside into the enzyme’s active site. Starting from *high anti* χ = –64° in 1XC8, the orientation of the oxoG base in all models spontaneously drifted towards the *anti* range, with 9 of 12 models remaining mostly in this range, with brief excursions into the *syn* domain (Fig. 5a). Two models, PRO-GLH-Aa and PRO-GLU-C, had reverted to *syn*, remaining in its *high anti* sub-range (χ = –83° ± 12°), whereas a single model, PRO-GLU-Aa, ventured deeper into the *anti* range (χ = –131° ± 11°; Fig. 5a).

In the *high syn* 1R2Y model of *Bst*-Fpg, four consecutive main chain amide nitrogens belonging to the Thr220–Tyr224 loop form a crown around O^6^ of oxoG, positioned at distances and angles suitable for hydrogen bond formation (Fig. 5c). Surprisingly, even though the oxoG base is rotated nearly 180° from its position in 1R2Y, the same set of contacts is maintained by the homologous loop Ser218–Tyr222 of *Lla*-Fpg (Fig. 5b, d). This observation agrees well with the literature data on simulation of *Bst*-Fpg with oxoG forced into the *anti* conformation [60] and with the same pattern of contacts to O^6^ in the 1XC8 structure of *Lla*-Fpg [23]. Notably, the “distinguishing” bond between the main chain carbonyl of Ser220 and pyrrolic N7 of oxoG, seen in *Bst*-Fpg 1R2Y structure but absent from *Lla*-Fpg 1XC8, was not observed in our simulations.

#### Interactions and dynamics of the opposite base

In all reported structures of Fpg bound to DNA with the fully everted damaged nucleotide, specific recognition of C opposite to the lesion is governed by two hydrogen bonds from Arg109 after its insertion into the DNA void: Nε[Arg109]–O^2^[C^(0)^] and Nη2[Arg109]–N3[C^(0)^]. If G substitutes for C, Nε and Nη2 of the inserted Arg form slightly suboptimal bonds with N7 and O^6^, respectively, of the G base in the *syn* orientation, whereas T in place of C retains a bond with Nε[Arg109] but experiences a clash between two hydrogen bond donors, N3[T] and Nη2[Arg109] [20].

**Fig. 6:**
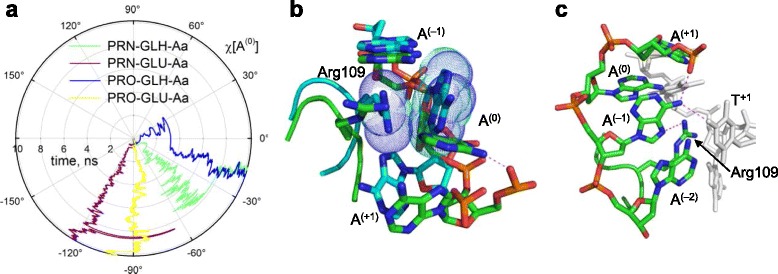
Conformation of the models around the orphaned nucleotide. **a,** glycosidic angle of the orphaned A^(0)^ nucleotide in the Aa models. **b,** overlay of structures from two snapshots at 6 ns (PRN-GLU-Aa model, carbons colored *green*; PRO-GLH-Aa model, *cyan*, the same structure as in Panel J but slightly turned for a clearer view) showing the central three bases (the non-damaged strand) and Arg109. The hydrogen bond between N6[A^(0)^] and O2P[A^(+1)^] in the PRO-GLH-Aa model and stacking between the partially extrahelical A^(0)^ and Arg109 in the PRN-GLU-Aa model are shown. **c,** structures from an 8-ns snapshot (PRO-GLH-As model) showing the central four base pairs and Arg109. The non-damaged DNA strand is colored. Note the hydrogen bonds formed by the orphaned A base with other nucleotides and Arg109

In all our C models, O^2^[C^(0)^] and N3[C^(0)^] maintained stable ~3 Å bonds with their interaction partners throughout the simulation (Additional file 1: Fig. S3A, B). In contrast, A^(0)^ in our *anti* simulations existed in two configurations. In both GLU-Aa models, it remained intrahelical in the *anti* orientation, stabilized by a hydrogen bond between its exocyclic N^6^ and the O2P non-bridging oxygen of A^(+1)^ (Fig. 6a, b and Additional file 1: Fig. S3C). In both GLH-Aa models, A^(0)^ is pushed towards the major groove and rotated halfway to the *syn* orientation, so it essentially lies extrahelically in the major groove with the Arg109 guanidine moiety stacked against A^(0)^ base (Fig. 6b). In the *syn* family of models, the A^(0)^ base was more stable. In all *syn* models, Nη2[Arg109] donated a hydrogen bond to N7[A^(0)^] (Additional file 1: Fig. S3D). The kinked conformation of DNA also allowed the exocyclic amino group of A^(0)^ to form additional non-canonical hydrogen bonds with other nucleotides: N^6^[A^(0)^]–O^4^[T^+1^] and N^6^[A^(0)^]–O2P[A^(+2)^] for a considerable part of the trajectories (Fig. 6c and Additional file 1: Fig. S3E, F). Considering the tendency of *anti* A^(0)^ to be expelled out of the stack, it is thus likely that in the Fpg-bound oxoG:A mispair (where, in the absence of the protein, A is *anti* [14, 15]), the orphaned A ultimately adopts *syn* conformation.

Despite the geometry of the As models is less disturbed in comparison with the Aa models, oxoG:A experimentally is still a poor substrate for Fpg. A comparison of equilibrium binding, steady-state and pre-steady state kinetics of the *E. coli* enzyme [4, 76] suggests that the Fpg–oxoG:A complex forms quickly but then is much slower to proceed to the catalytically competent conformation than the Fpg–oxoG:C complex, leading to a ~15-fold higher apparent *K*_M_ (14 nM for oxoG:C vs 190 nM for oxoG:A [4]; 8.7 nM for oxoG:C vs 150 nM for oxoG:A [76]). At the same time, the observed effect of A on *k*_cat_ is minor [4, 76]. Since *K*_M_ reflects the population of the last pre-catalytic state, it is tempting to suggest that only oxoG:A(*syn*) may be capable of attaining the catalytically competent conformation, thus necessitating the *anti*–*syn* transition in the course of the productive reaction with oxoG:A and partly explaining its slow progression with this substrate where A is initially *anti* [14, 15].

#### Aromatic wedge-induced distortion

**Fig. 7:**
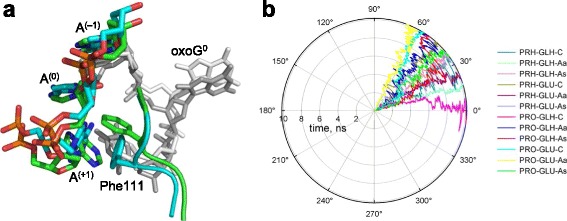
**a,** overlay of the structures (PRN-GLU-C model) illustrating the retraction of the intercalating side chain of Phe111. The structure with carbons colored *green* is the starting structure after minimization (0 ns); the structure with carbons colored *cyan* is at 8 ns. The protein backbone (residues 109–113) is shown in the cartoon representation, colored in the same way, with Phe111 presented as a stick model. The N, O, and P atoms are colored *blue*, *red*, and *orange*, respectively. In DNA, only the non-damaged strand is colored. Note the protein backbone movement, accompanied with ~90° Phe111 ring turn, and the corresponding turn of A^(+1)^ to keep the phenyl ring stacked with the purine heterocycle. **b,** propeller twist angle (ω) of the pair T^–1^:A^(+1)^. In B-DNA (PDB ID 355D) [80], ω = 13° ± 4°

In all known structures of Fpg bound to DNA, a Phe residue (Phe111 in *Lla*-Fpg) is inserted between the sampled base pair and the base pair 3′ to it (Fig. 1c). The sampled pair is significantly buckled but the strain is relieved upon everting the damaged base, with the Phe wedge remaining to contact the orphaned base and its neighbor in the undamaged strand [25, 77]. Interestingly, in the structures of *Bst*-Fpg containing C, T or G opposite a reduced AP site, the Phe wedges overlap almost perfectly [20]. However, in our models, the Phe residue showed significant mobility: in 8 out of 12 models, Phe111 retreated back into the minor groove. This movement was accompanied with a significant turn of the A^(+1)^ base, which maintained stacking with Phe111: in 9 out of 12 models, the area of contact between the Phe111 side chain and the adenine was larger than in 1XC8 for more than half of the simulation (Fig. 7a and Additional file 1: Fig. S4). As a result, the T^–1^:A^(+1)^ pair was grossly distorted, mostly by the propeller twist movement (Fig. 7a, b). In the remaining four models, one (PRO-GLH-C) displayed brief aborted attempts to retract Phe in the same manner (with full retraction in one of the repeats), in one (PRO-GLH-Aa), the A^(+1)^ moved by a buckling motion allowing Phe to unstack and adopt an alternative conformation without leaving the double helix, and only in two models (PRN-GLH-C and PRN-GLU-Aa) the initial conformation of the wedge and the adjacent nucleotides was stable.

### Specific distant interactions in Fpg–DNA complexes

#### Model-specific hydrogen bonds

In order to select out inter- and intramolecular interactions specific for oxoG:C, we have searched for hydrogen bonds that existed (i. e., had an energy > 1.2 kcal/mol) in > 1% of the snapshots. Around 600 such hydrogen bonds were found in each model. In all models, less than 50% of the found bonds existed for more than 90% of the snapshots, and less than 25% of the found bonds existed in less than 25% of the snapshots (Additional file 1: Fig. S5A, B). The former class may be considered to represent stable, functionally important hydrogen bonds, whereas the latter one is most likely due to conformational fluctuations. Therefore, all detected hydrogen bonds were first analyzed with respect to their occurrence in these categories (≤90% vs > 90% and ≤ 25% vs > 25%). Pearson’s mean square contingency coefficients (φ) for pairwise comparison between different models showed no significant contribution of protonation state or substrate into the overall distribution of bonds in the high- and low-stability categories.

**Fig. 8:**
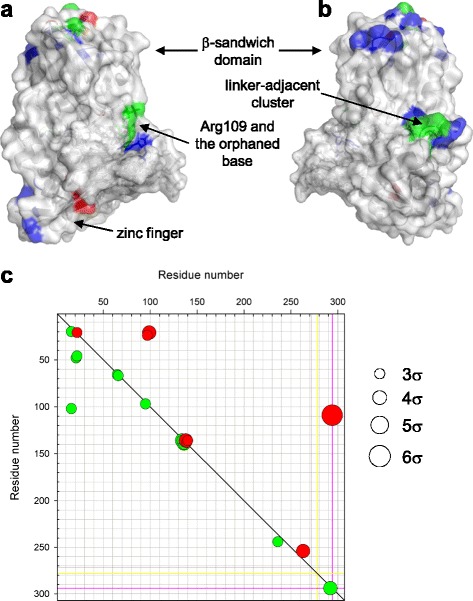
**a,** surface representation of the PRO-GLH-C model (8 ns) showing parts of the molecule where C/A-specific hydrogen bonds are found. Residues forming C-specific bonds only are colored *red*, those forming exclusively A-specific bonds are *blue*, and the residues forming alternative bonds in C and A models are *green*. **b,** the same model as in **a** rotated 180° around the vertical axis. **c,** interaction difference map showing pairs of hydrogen bond-forming amino acids specific (>3σ difference in bond occurrence calculated over all pairs of models) for C models (*red*) or A models (*green*). Residues 1–271, protein; 272–285, damaged DNA strand; 286–299, complementary DNA strand; 300, Zn^2+^; 301–307, structural water molecules. The *yellow* line marks the position of oxoG^0^, the *magenta* line, the position of C^(0)^/A^(0)^

We then searched for the bonds that were consistently different between C, Aa, and As models, selecting those deviating > 3σ from the mean distance between the models (Fig. 8 and Additional file 1: Fig. S6A–C). Only a few bonds consistently showed different stability in all C *vs* A comparisons irrespective of the *syn* or *anti* conformation of A^(0)^. Unsurprisingly, some of these were formed by the orphaned base itself (Fig. 8). Notably, the oxoG nucleotide, the O^6^-binding crown loop, and the Pro1–Glu2 catalytic dyad formed no model-specific bonds. Moreover, a comparison of bonds specific for protonation states (PRO *vs* PRN, GLU *vs* GLH) revealed only a few isolated bonds remote from the active site (Additional file 1: Fig. S6D, E).

#### Fpg regions with C-specific bonds outside the active site

The most prominent opposite-base-specific feature in the protein structure was a cluster at the start of the C-terminal domain immediately next to the interdomain linker (residues Glu134–Phe140). Most of the amino acid residues there engaged in multiple bonds, forming a network, which existed in two stable configurations. In one, which was statistically significantly more often observed in A models, Thr136 formed two bonds with Glu134, one with Asp139, and one with Phe140 (N[Thr136]–O[Glu134], Oγ[Thr136]–O[Glu134], N[Asp139]–Oγ[Thr136], N[Phe140]–Oγ[Thr136]), and a N[Tyr137]–Oε1/Oε2[Glu138] was present. A completely different set of bonds was characteristic of C models (Oγ[Thr136]–Oε1/Oε2[Glu138], N[Asp139]–Oε1/Oε2[Glu138], N[Phe140]–O[Thr136]). As a result, the Glu134–Phe140 loop adopted different conformations in the C and A models (Fig. 8). Importantly, the conservation of Fpg sequence is quite high in this region (Additional file 1: Fig. S7), underlying its functional significance despite its position well away from the active site.

The only other region of known functional importance where consistently different bonds existed was the β-hairpin zinc finger, a structural motif in Fpg involved in major groove tracking and lesion recognition [35] (Fig. 8). Several C/A-specific hydrogen bonds were scattered in the β-sandwich domain around the C-terminal end of the long α-helix αA, which carries the catalytic Pro–Glu dyad at the other end (Fig. 8). The functional significance of this region is not clear; most C/A specific residues here are located in surface loops and are not conserved (Additional file 1: Fig. S7).

#### Fpg regions with A(syn) and A(anti)-specific bonds outside the active site

In addition, we have searched for bonds specific for A models in different (*anti* or *syn*) conformations of the orphaned A (Additional file 1: Fig. S6C). Most of the differences were encountered between protein and DNA, and within DNA, reflecting the conformational changes inflicted by introducing the disfavored A base. The protein residues affected by the conformation of the orphaned nucleotide showed little overlap with the C/A-specific interactions. The most prominent Aa/As-specific contacts were formed by Tyr29/Arg31 and His91/Lys110, two elements that coordinate the phosphates flanking the orphaned A, and Lys155 that contacts DNA a few nucleotides away from the lesion but is important for Fpg activity [6]. The Glu134–Phe140 C/A-specific linker-adjacent region showed no significant difference between Aa and As models.

### Water molecules in the Fpg–DNA complex

#### Dynamics of structural water in Fpg

The structure of *Lla*-Fpg–DNA complex, 1XC8, contains the total of 397 water molecules. However, only 22 of those reside at the protein–DNA interface and only seven are buried at it (i. e., have < 10% solvent exposure). The structures of Fpg–DNA complexes from different species, as well as the structures of the homolog of Fpg, *Eco*-Nei, in a complex with DNA [78], suggest that several water molecules form a tight network of bonds in the enzyme’s active site that may serve to shuttle protons during the concerted cleavage of three bonds catalyzed by Fpg.

We have explicitly modeled the seven water molecules buried at the protein–DNA interface and determined whether they form hydrogen bonds with two or three Fpg or DNA donors or acceptors at the same snapshot. Such water bridges, if persistent, may indicate an important role of water in structure maintenance or reaction mechanism. There were no significant differences between models or between groups of models in the number of water bridges. One particular pair of acceptors, Oε1/Oε2[Glu76] and O^8^[oxoG^0^], was consistently found bridged by two water molecules in 8 of 12 models. In several models, such multiple water-mediated connections existed between the non-bridging phosphate oxygens of oxoG^0^ and T^+1^ and between O2P[oxoG^0^] and Nη1[Arg109] but their occurrence was much less common. No donor/acceptor triplets were connected by multiple bridges.

**Fig. 9:**
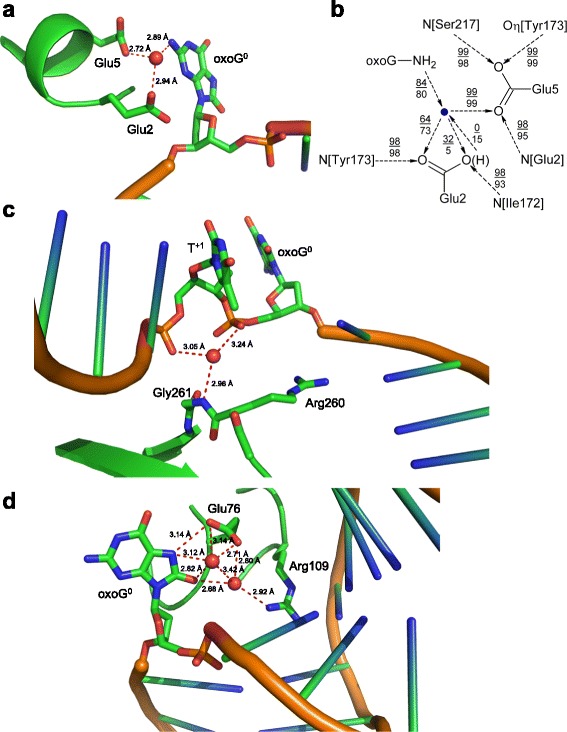
View of the PRO-GLH-C model (8 ns, the same snapshot as in Fig. 8) showing water traps. Protein and DNA residues coordinating the water molecule (*red ball*) are shown as a stick model and colored according to atom type (*green*, C; *blue*, N; *red*, O; *orange*, P). Other parts of the complex are either shown as a cartoon model or hidden for clarity. Distances between possible hydrogen bond donors and acceptors are indicated by dashed lines. **a,** Glu2, Glu5, and oxoG^0^. **b,** schematic representation of hydrogen bonds formed by the water molecule (*blue dot*) trapped between Glu2, Glu5, and oxoG^0^. The numbers indicate percentage of snapshots in which the bond is observed, averaged over all GLU models (top) or GLH models (bottom). **c,** Arg260, Gly261, oxoG^0^ and T^+1^. **d,** Glu76, Arg109, and oxoG^0^

In order to single out the preferred sites of water binding in the Fpg–DNA structure, we have looked in more detail at the water bridges with the occurrence above a threshold of 2000 (for pairs) or 1500 (for triplets). These thresholds cut off the lowest quartile of the cumulative distribution of bridges averaged over all twelve models, i.e., they define the bridges that collectively account for >75% of all occurrences (Additional file 1: Fig. S8). The most frequent triplet was formed by Oε1/Oε2[Glu2], Oε1[Glu5] and N^2^[oxoG^0^] (Fig. 9a); it was found in the high range in 11 out of 12 models and was not far below the 1500 cut-off (1273) in the remaining one (PRO-GLH-Aa). A cluster of spots habitually occupied by a water molecule was near Nε[Arg260] and N[Gly261] in the protein and non-bridging oxygens of oxoG^0^ and T^+1^ in DNA (Fig. 9c). Usually, a single water molecule was found in this region at any one snapshot, alternating between different triplets of donors and acceptors. Finally, Oε1/Oε2[Glu76] formed triplets with Nη1[Arg109] and O^2^[T^+1^] or O^8^[oxoG^0^] (9 out of 12 models in total) with two water molecules involved (Fig. 9d). Other triplets, even those passing the threshold of 1500, were found in 1–3 models and are not expected to be significant.

#### Possible role of structural water molecules in Fpg mechanism

The identified stable triplets are suggestive of an important role of water in the mechanism of action of Fpg. The water molecule trapped between Oε1/Oε2[Glu2], Oε1[Glu5] and N^2^[oxoG^0^] is located at the position suitable for proton transfer to Glu2, required for the protonation of O4′ of oxoG nucleotide; water-mediated proton transfer to Glu2 was earlier proposed on structural reasons [19, 26]. In the GLH models, this water stably donated a bond only to the unprotonated Oε1[Glu2] (73% bond occurrence averaged over all GLH models, compare with 5% for the bond to Oε2) but when Glu2 was charged, Oε2 accepted this hydrogen with a higher frequency (64% and 32% bonds to Oε1 and Oε2, respectively) (Fig. 9b). In the GLH-Aa models, the protonated Oε2 showed a tendency to donate a hydrogen bond to the water molecule rather than accept one (23% in PRN-GLH-Aa, 58% in PRO-GLH-Aa, 0–7% in other GLH models), consistent with poor substrate properties of *anti* A. It should be mentioned that in QM/MM analysis of fapyG excision by Fpg this water molecule was inhibitory to the reaction, preventing the protonation of O4′ by neutral Glu2 [36] and should be displaced from its crystallographic position after donating a proton to Oε2.

The water molecule bridging the protein residues with the phosphates of T^+1^ and oxoG^0^ may be important for distorting the DNA duplex. Notably, the distance between the phosphorus atoms P[T^+1^] and P[oxoG^0^] is significantly shorter than in the regular B-DNA in all models. This pinching of the phosphates around T^+1^, together with wedging of Phe111 and insertion of Met75 and Arg109, assists in kinking the DNA axis by ~60° and eversion of the damaged nucleotide.

The tightly coordinated two-water bridge to O^8^[oxoG^0^] presents an intriguing conundrum. On one hand, water-mediated recognition of this unique carbonyl would be an attractive mechanism of direct oxoG sensing in the active site pocket. On the other hand, Glu76, which in our models participates in the water coordination, is present only in a small branch of the Fpg family tree consisting of two closely phylogenetically related groups, Bacilli and Mollicutes (which include *L. lactis* and *G. stearothermophilus*), while in all other Fpg sequences this position is occupied by Ser/Thr with very rare exceptions (Additional file 1: Fig. S9, Additional file 3). In the structure of *Bst*-Fpg, the presence of Glu76 stabilizes the everted oxoG in the *high syn* conformation through hydrogen bonding with N^2^[oxoG], whereas its *in silico* replacement with Ser reverts the preferred χ angle to the *anti* domain [60]. In *Eco*-Fpg, Ser74 and Lys217 correspond to Glu76 and Arg220 of *Lla*-Fpg, and Lys217 forms a direct hydrogen bond with O^8^[oxoG] [38]. Obviously, there are several ways by which Fpg enzymes can employ the residues at these positions to the effect of recognizing the exocyclic oxygen at C8 either directly or indirectly.

## Conclusion

The opposite-base selectivity of Fpg and some other DNA glycosylases (eukaryotic OGG1 and TDG, bacterial MutY and Mug, etc.) is extremely important for the prevention of mutations in the course of DNA repair. Analysis of the causes of this selectivity is complicated due to the paucity of structures of DNA glycosylases bound to substrates with disfavored opposite bases. In the case of Fpg, no structure containing oxoG:A, the biologically relevant disfavored mispair, is available. Our modeling effort was mostly undertaken to analyze possible structural features of such a complex and reveal those that could explain low activity of Fpg on oxoG:A substrates.

As our models suggest, introduction of A opposite to oxoG indeed distorts the protein–DNA interface within ±2 base pairs around the lesion site, outside of which DNA exists as a normal duplex. Arg-109 and Phe-111, two residues that Fpg inserts into DNA to sharply kink it and maintain oxoG everted from the base stack, tended to withdraw if A was opposite to the lesion, indicating that the pre-catalytic complex of Fpg with oxoG:A is inherently unstable. Interestingly, although the oxoG:A mispair adopts oxoG(*syn*):A(*anti*) conformation in free DNA, our models showed that upon Fpg binding and oxoG eversion, the orphaned A is more stable as a *syn* conformer, engaged both in hydrogen bonding with Arg-109 and in base stacking. We speculate that Fpg binding to oxoG(*syn*):A(*anti*) may be energetically disadvantageous and require rotation of the A base around the glycosidic bond for rare events of base excision; direct test of this hypothesis would require solving the structure of Fpg–DNA(oxoG:A) complex or stopped-flow kinetics with a series of fluorescent reporter bases incorporated next to A, in which case the *anti–syn* transition may be expected to be observed in the fluorescent traces.

Analysis of model-specific hydrogen bonds unexpectedly revealed a cluster of highly conserved residues next to the interdomain linker of Fpg, which adopted alternative conformations when C or A was in the opposite strand. This cluster is remote from what is usually considered the active site of Fpg; however, it is packed against a helix–two-turn–helix motif that is present in all Fpg family members and partly forms the DNA-binding groove. Of note, it has been shown that in Nei, a homolog of Fpg specific for oxidized pyrimidines, a structural rearrangement of the linker and the region adjacent to it induces productive DNA binding [79]. Thus, our models add weight to a hypothesis of indirect readout by DNA glycosylases, which states that recognition of damaged bases is not limited to formation of specific bonds but greatly relies on the differences in energetics and dynamics of protein and DNA parts that may be far away from the moiety being recognized.

Structural and kinetic data together with QM/MM modeling of Fpg favor the reaction chemistry that combines a nucleophilic attack at C1′ of oxoG by N[Pro1] residue and protonation of O4′ of oxoG by Oε2[Glu2] [33, 35, 36]. The latter step is important since it affords a ~60 kcal/mol lower barrier to glycosidic bond breakage compared to base protonation as the leaving group activation [33]. Such mechanism requires Pro1 to be in the unprotonated, and Glu2, in the protonated state immediately before the reaction, implying that both these residues should change their preferred protonation state. Our measurements of the pH dependence of Fpg activity suggest that only one group is ionized in a pH-dependent manner, in which case it is consistent with Pro1 N-terminal secondary amine losing a proton at increasing pH. Consequently, the ionization state of Glu2 in the Fpg–DNA complex shows no evidence of being pH-dependent, which means that the assembled active site is capable of protonating Glu2, possibly using a water molecule as a proton shuttle. The arrangement of the reacting atoms is only consistent with the reaction stereochemistry with S_N_2 displacement of O4′ as the first step, in agreement with the QM/MM data [33]. Since the substitution of Gln for Glu2 inactivates the enzyme, which rules out simple hydrogen bonding as the primary function of Glu2, the mechanistic implication of our results is that Glu2 has to be deprotonated again later in the reaction, likely by the nascent alkoxide O4′, and contribute its charge to the stabilization of the transition state of the departing oxoG base. In the QM/MM simulation, several consecutive acts of proton transfer between Oε2[Glu2], O4′[oxoG], N[Pro1], and O^8^[oxoG] allow the enzyme to lower the highest barrier in the reaction from 71 kcal/mole (as with direct oxoG protonation path) to 13 kcal/mole relative to the lesion recognition complex [33]; a similar energy gain was calculated for fapyG excision [36].

Finally, our modeling concerned only the pre-catalytic complex of Fpg–DNA. It is now clear that the selectivity of DNA glycosylases is not determined exclusively by interactions in their pre-catalytic complexes, the structures of which are relative easy to establish by X-ray crystallography, but also relies on several kinetic gates along the full recognition pathway, including primary encounter and damaged base eversion. Future modeling of the early steps of recognition of oxoG-containing pairs will add clarity to our understanding of the opposite-base discrimination by Fpg.

## Additional files

Additional file 1: Figure S1. **A,** R.m.s.d. of the models over time. The traces are color-coded: dark *cyan*, PRN-GLH-C; l*ight lime*, PRN-GLH-Aa; *coral*, PRN-GLH-As; *olive*, PRN-GLU-C; dark *magenta*, PRN-GLU-Aa; *light blue*, PRN-GLU-As; *magenta*, PRO-GLH-C; *blue*, PRO-GLH-Aa; *red*, PRO-GLH-As; *cyan*, PRO-GLU-C; *yellow*, PRO-GLU-Aa; *green*, PRO-GLU-As. **B,** Reproducibility of the repeat runs. R.m.s.d. of the initial run (*red*) and three repeat runs (*green*, *blue*, and *magenta*) of the PRO-GLH-C model are shown together with the cross-run r.m.s.d. between two pairs of the repeat runs (*black* and *green*). Repeat runs of other models produced similar within-run and cross-run r.m.s.d. values and are not shown. **Figure S2.** Circular dichroism spectrum of Fpg at pH 4.0 (*black circles*) and pH 7.6 (*white circles*). **Figure S3.** Conformation of the models around the orphaned nucleotide. **A,** distance Nε[Arg109]…O^2^[C^(0)^] in the C models. **B,** distance Nη2[Arg109]…N3[C^(0)^] in the C models. **C,** distance N^6^[A^(0)^]…O2P[A^(+1)^] in the Aa models. **D,** distance Nη2[Arg109]…N7[A^(0)^] in the As models. **E,** distance N^6^[A^(0)^]…O4[T^+1^] in the As models. **F,** distance N^6^[A^(0)^]…O2P[A^(+2)^] in the As models. Moving average of a 50-snapshot window is shown in all panels. **Figure S4.** Occluded area (inaccessible to a 1.4 Å probe) between Phe111 side chain and A^(+1)^ base. The colors of the traces are the same as in Fig. S1. The dashed line indicates the occluded area in the 1XC8 structure. Moving average of a 50-snapshot window is shown. **Figure S5. A,** Cumulative distribution of the occurrence of hydrogen bonds in the *Lla*-Fpg–DNA complex. **B,** Overall reproducibility of hydrogen bonds in replicate PRO-GLH runs. Dots show the coefficient of variation for the occurrence of a particular hydrogen bond calculated over four replicates plotted against the mean occurrence of the bond. The histograms show the distribution of the mean occurrence. The scale in all panels is the same. Numbers above the graphs indicate the percentage of hydrogen bonds with the mean occurrence >90%. **Figure S6.** Interaction difference maps showing pairs of hydrogen-bond forming amino acids specific (>3σ difference in bond occurrence calculated over all pairs of models) for: **A**, C models (*red*) or Aa models (*blue*); **B**, C models (*red*) or As models (*cyan*); **C**, As models (*cyan*) or Aa models (*blue*); **D**, PRO models (*red*) or PRN models (*blue*); **E**, GLH models (*red*) or GLU models (*blue*). Larger circles correspond to larger deviations from the mean occurrence. Residues 1–271, protein; 272–285, damaged DNA strand; 286–299, complementary DNA strand; 300, Zn^2+^; 301–307, structural water molecules. The *yellow* line marks the position of oxoG^0^, the *magenta* line, the position of C^(0)^/A^(0)^. **Figure S7.** Conservation of Fpg sequence. **A,** plot of conservation number *C*_*n*_ against the residue position. **B,** view of the PRO-GLH-C model (8 ns) colored according to *C*_*n*_. **C,** the same model as in **B** rotated 180° around the vertical axis. The orientation of the molecule in **B** and **C** is the same as in Fig. 8. **Figure S8.** Rank plot of water-mediated bridges (top 100 occurrences) in the Fpg–DNA structures (**A–L**, the model nature is indicated in the respective panels). *Red*, pairs; *blue*, triplets. Dashed lines indicate cutoffs of 2000 snapshots for pairs and 1500 snapshots for triplets. Insets show cumulative distribution frequencies of pairs and triplets. **Figure S9.** Cladogram of Fpg sequences. The tree was constructed as described in Methods and visualized using TreeDyn [81]. (PDF 3904 kb)

Additional file 2: Table S1. p*K*_a_ of Pro1 and Glu2 in selected Fpg structures. (DOC 42 kb)

Additional file 3: Alignment of 124 sequences from the Fpg family. See Methods for sequence selection and alignment details. (TXT 101 kb)
