# Supplementary figures and images for: Molecular dynamics simulation of the opposite-base preference and interactions in the active site of formamidopyrimidine-DNA glycosylase
Source: BMC Struct Biol. 2017 May 8;17:5. doi: 10.1186/s12900-017-0075-y (PMC5422863; doi:10.1186/s12900-017-0075-y)

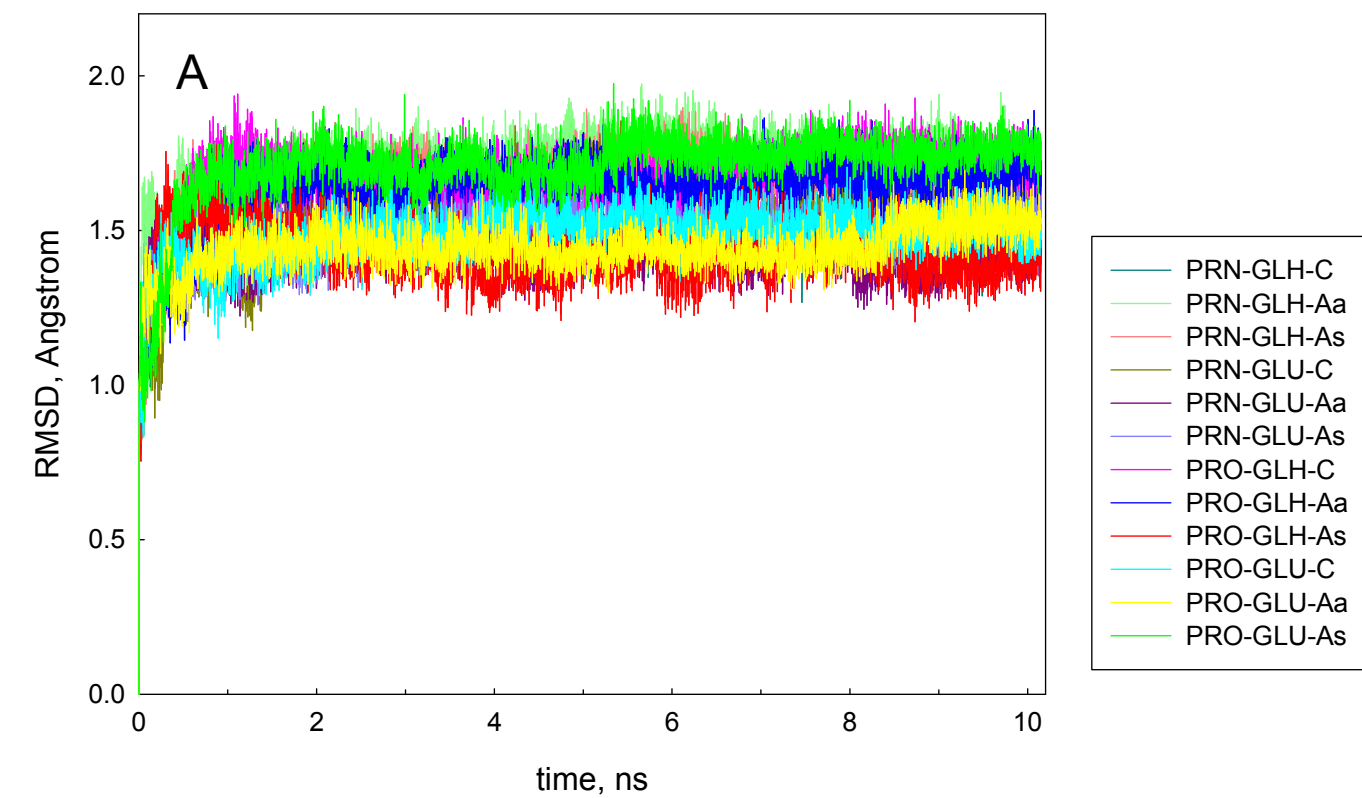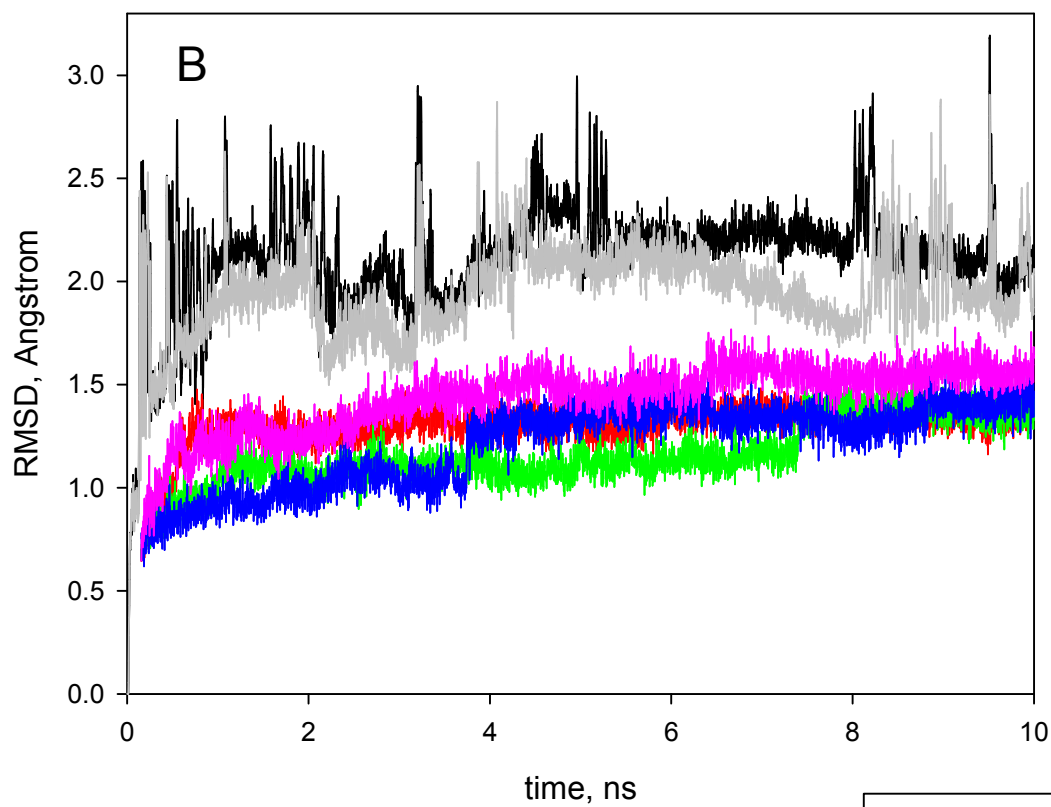

Fig. S1

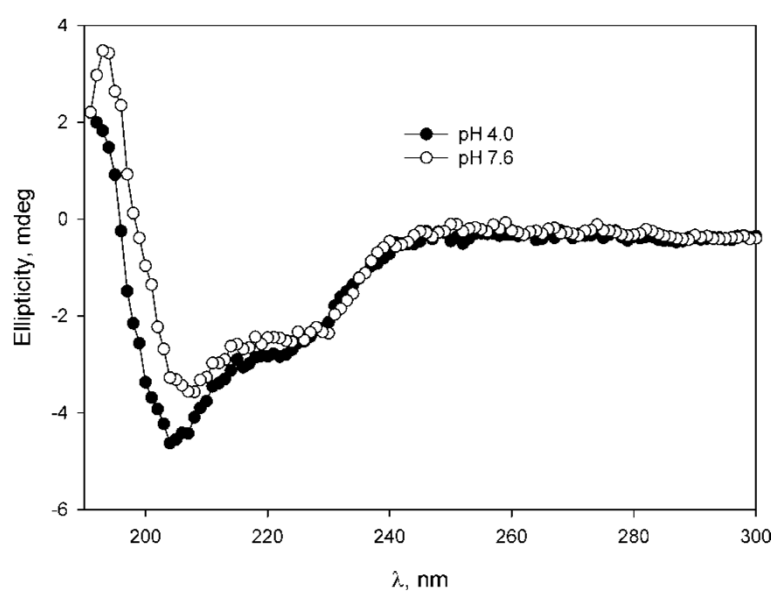

Fig. S2

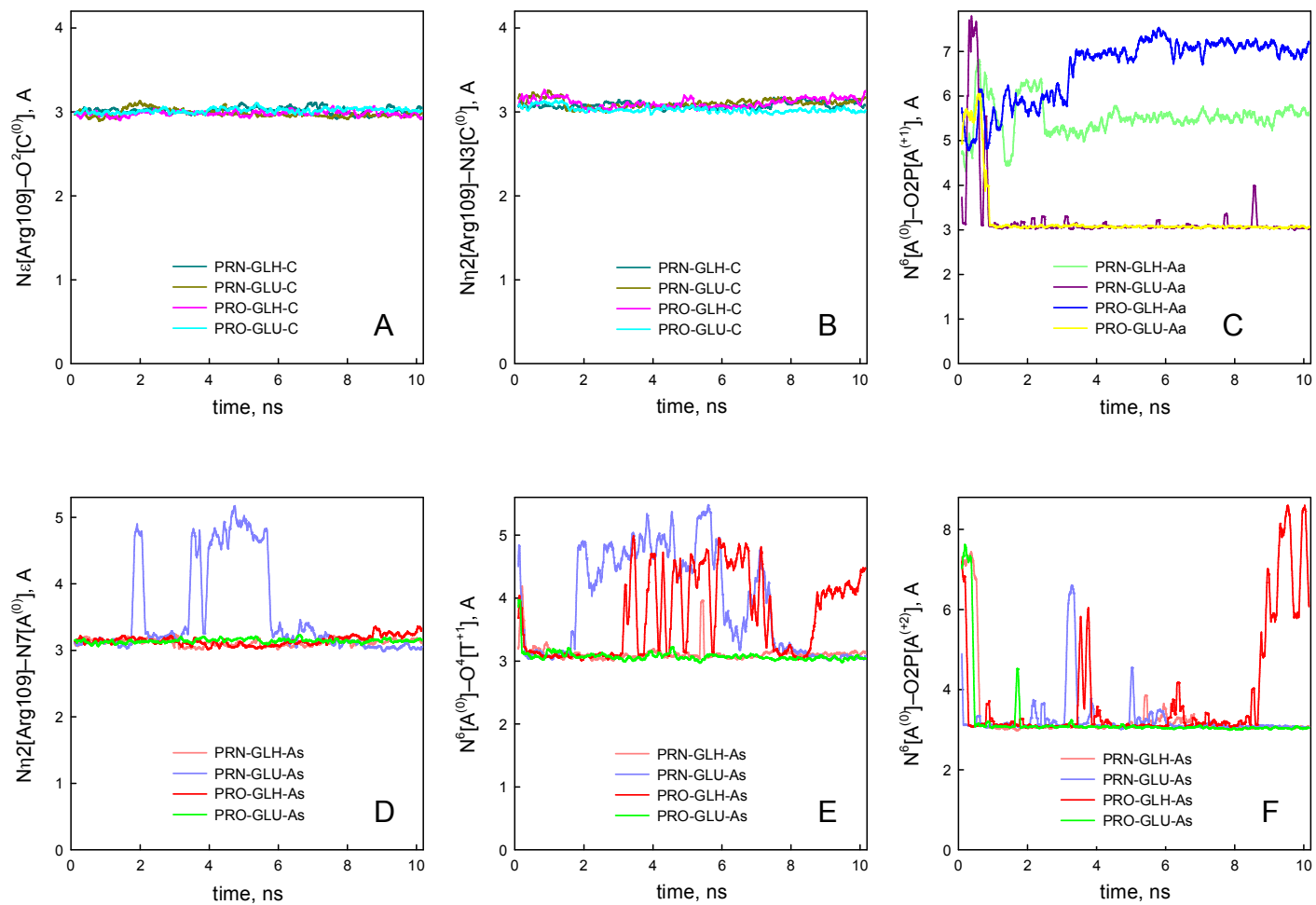

Fig. S3

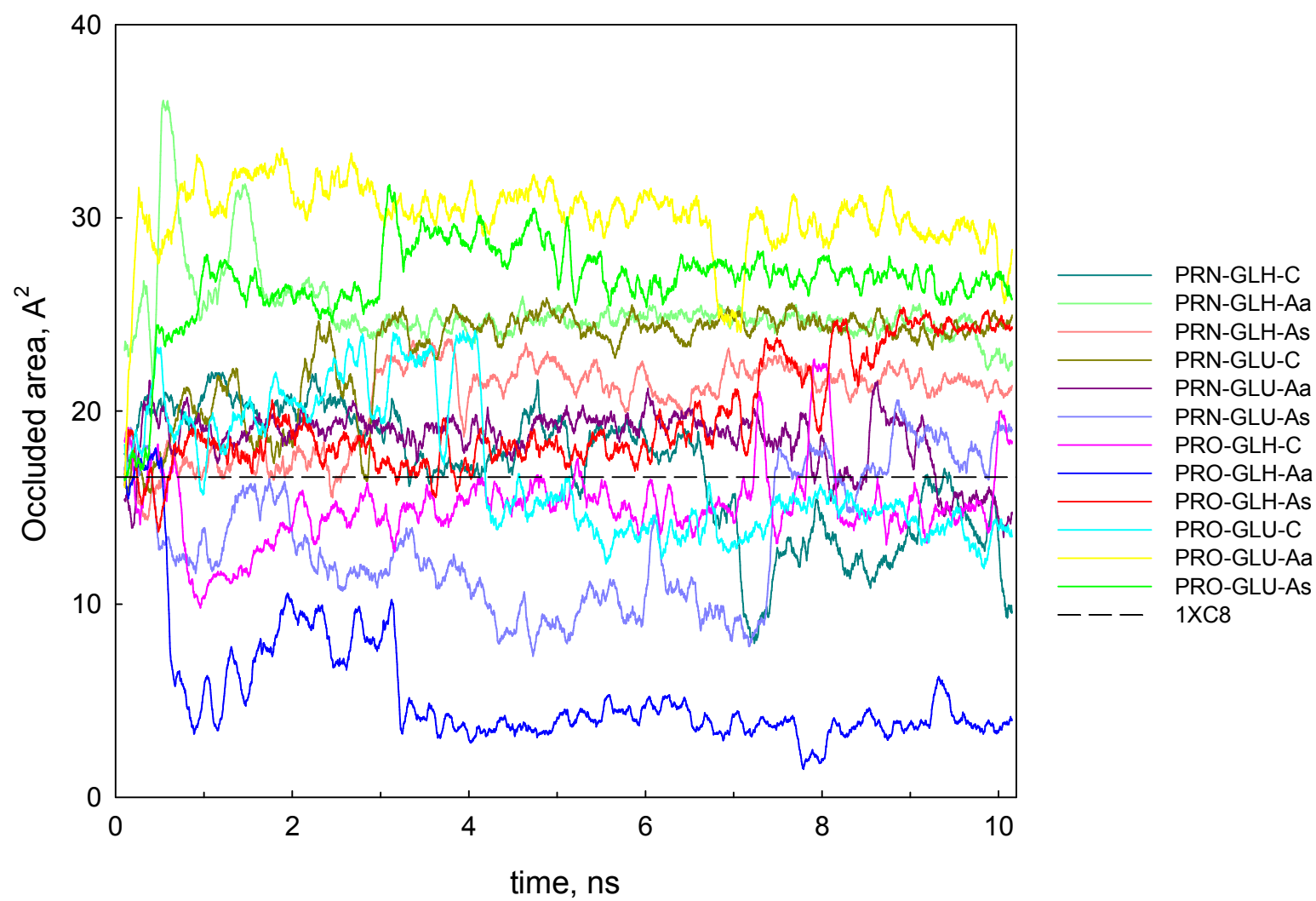

Fig. S4

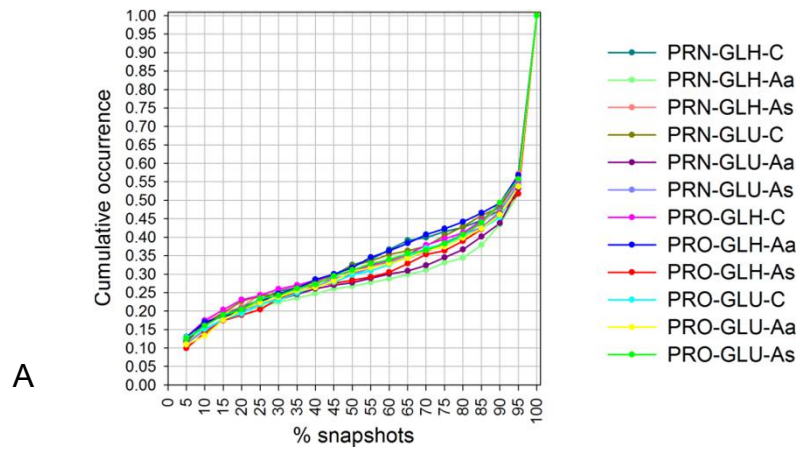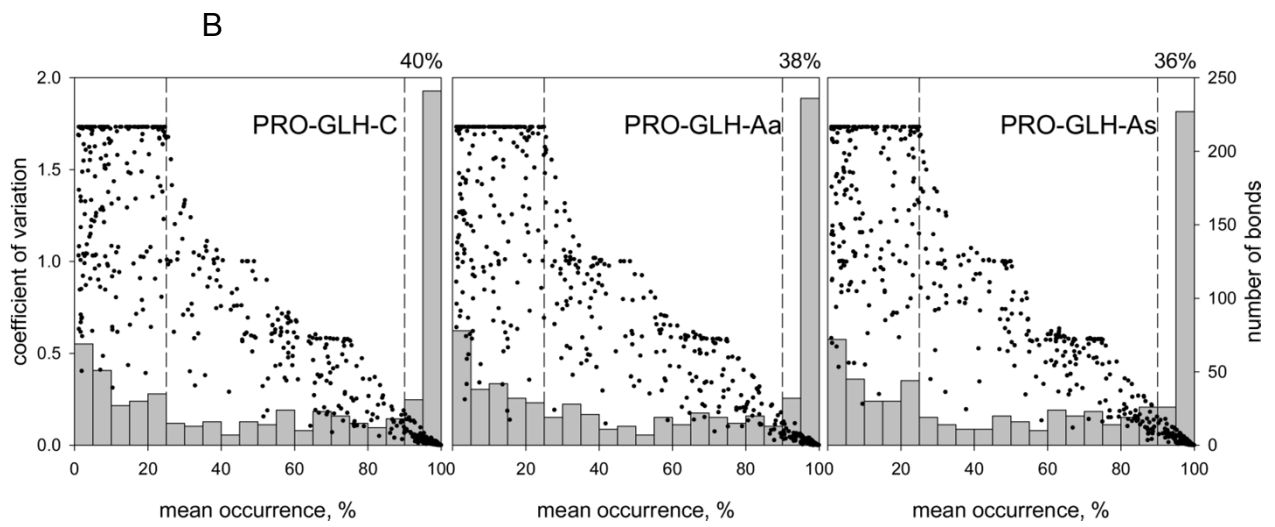

Fig. S5

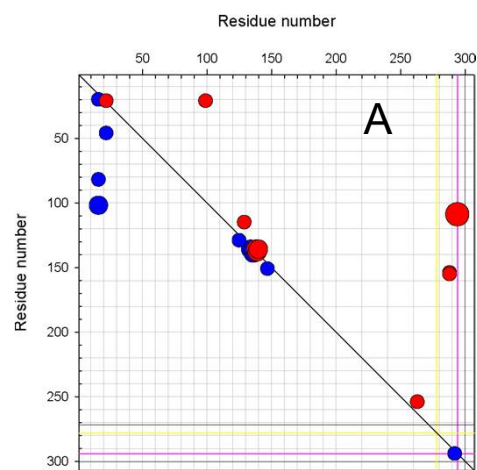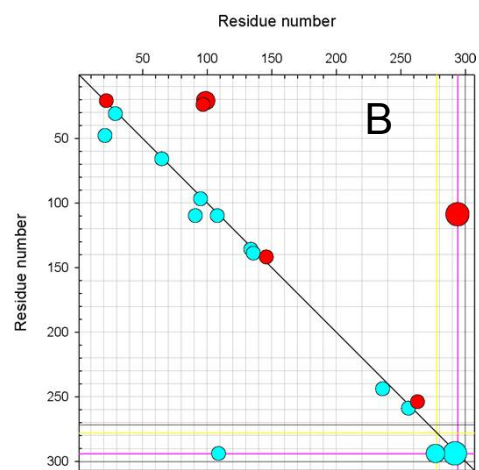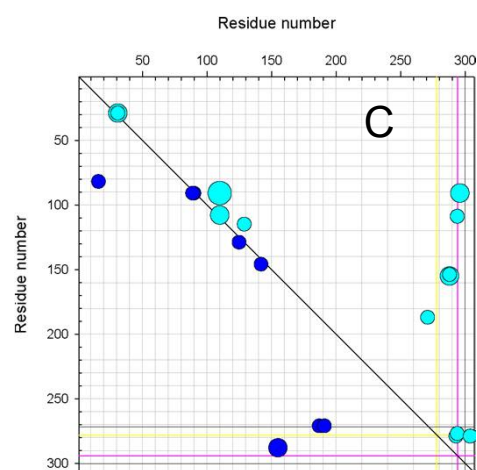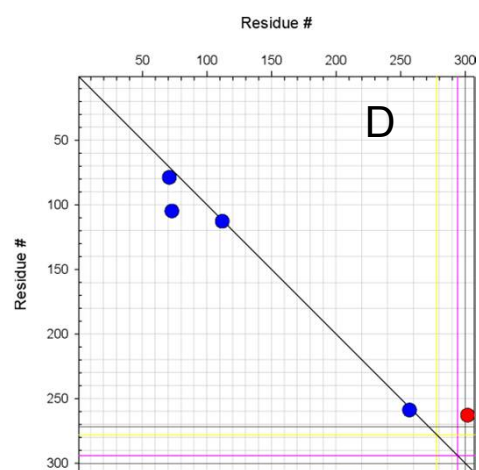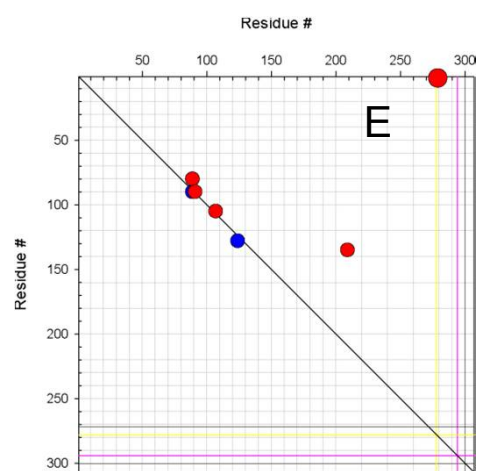

○  $3\sigma$   
○  $4\sigma$   
○  $5\sigma$

Fig. S6

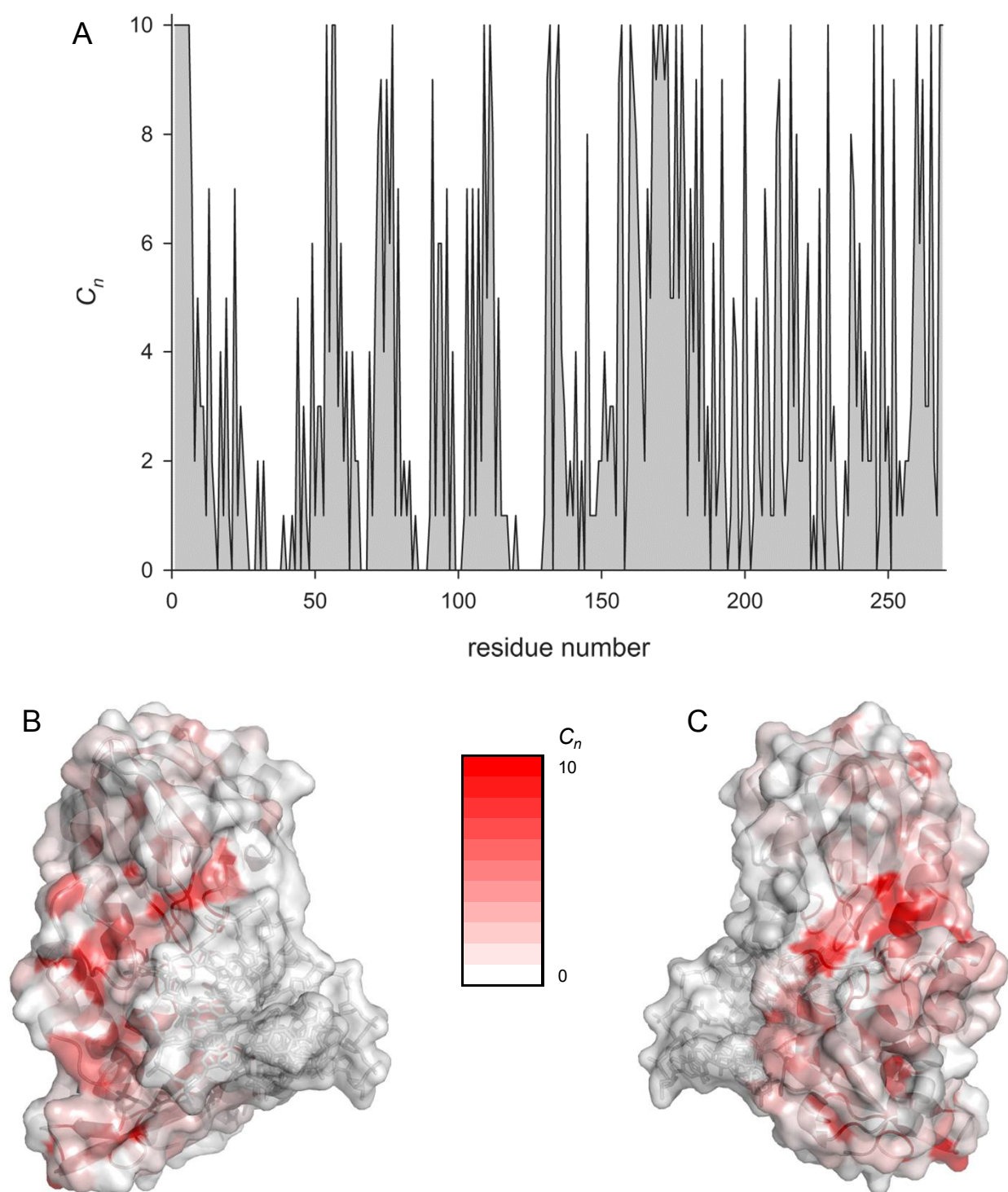

Fig. S7

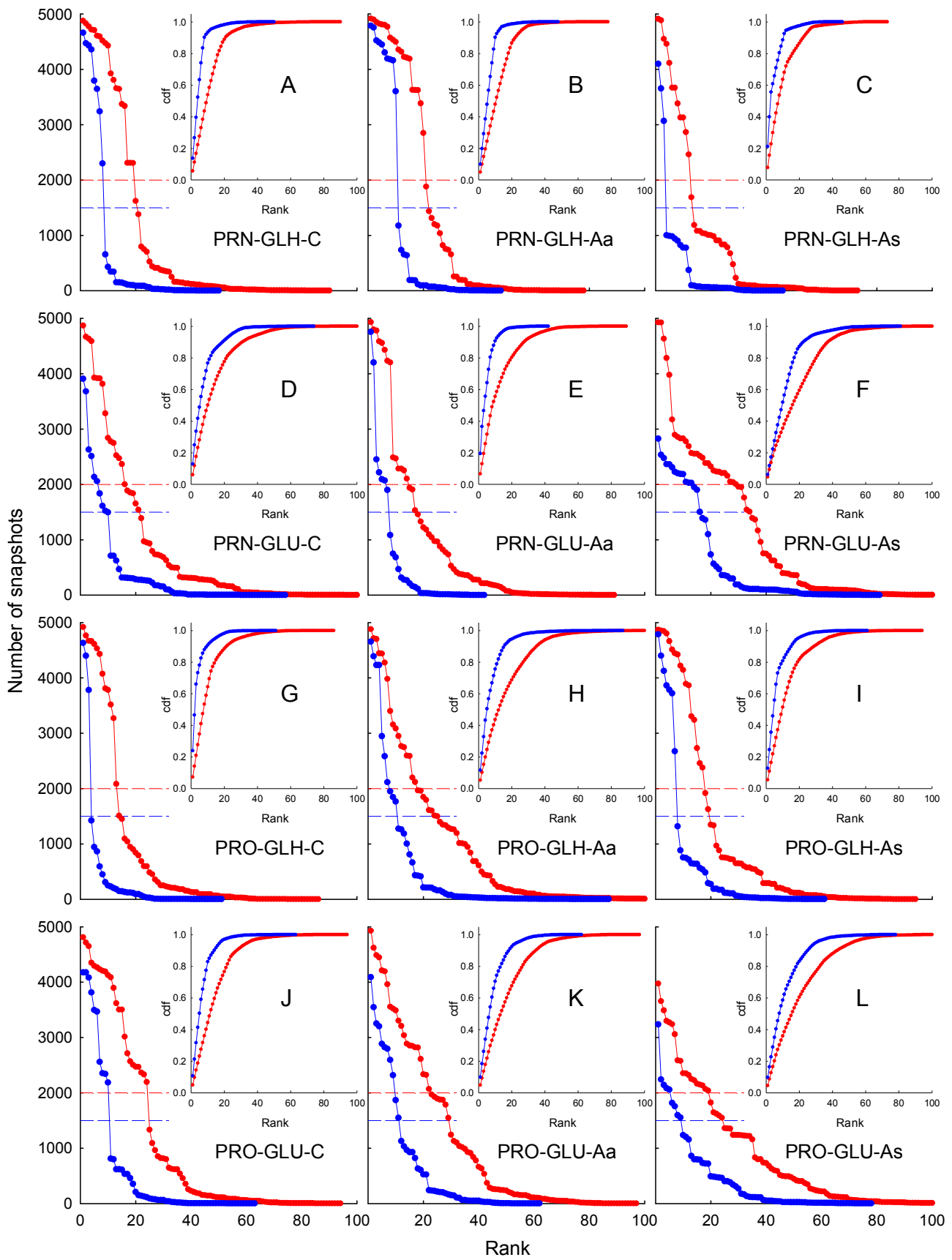

Fig. S8

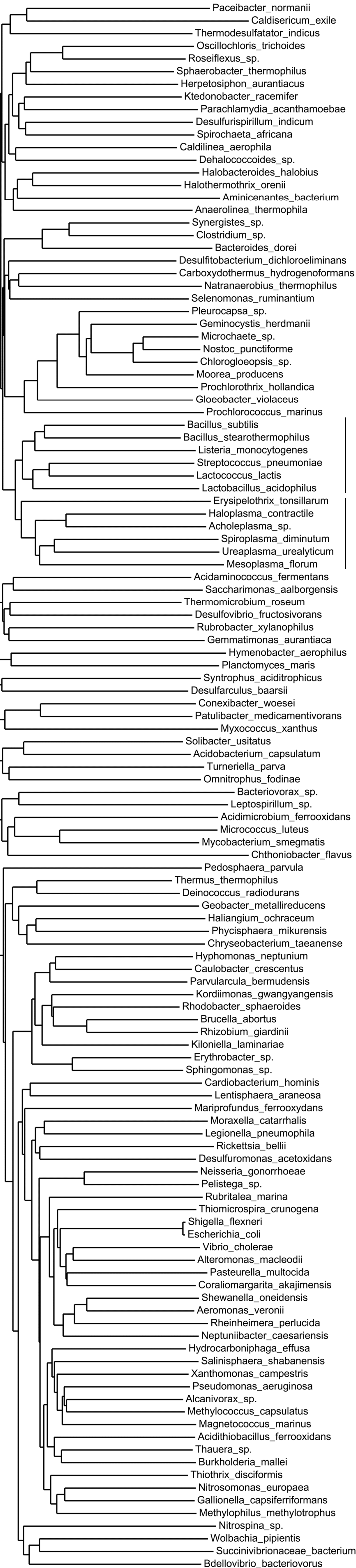

Mollicutes    Bacilli

Glu76

Fig. S9

Supplement: Supplementary file 1 — A, R.m.s.d. of the models over time. The traces are color-coded: dark cyan, PRN-GLH-C; light lime, PRN-GLH-Aa; coral, PRN-GLH-As; olive, PRN-GLU-C; dark magenta, PRN-GLU-Aa; light blue, PRN-GLU-As; magenta, PRO-GLH-C; blue, PRO-GLH-Aa; red, PRO-GLH-As; cyan, PRO-GLU-C; yellow, PRO-GLU-Aa; green, PRO-GLU-As. B, Reproducibility of the repeat runs. R.m.s.d. of the initial run (red) and three repeat runs (green, blue, and magenta) of the PRO-GLH-C model are shown together with the cross-run r.m.s.d. between two pairs of the repeat runs (black and green). Repeat runs of other models produced similar within-run and cross-run r.m.s.d. values and are not shown. Figure S2. Circular dichroism spectrum of Fpg at pH 4.0 (black circles) and pH 7.6 (white circles). Figure S3. Conformation of the models around the orphaned nucleotide. A, distance Nε[Arg109]…O2[C(0)] in the C models. B, distance Nη2[Arg109]…N3[C(0)] in the C models. C, distance N6[A(0)]…O2P[A(+1)] in the Aa models. D, distance Nη2[Arg109]…N7[A(0)] in the As models. E, distance N6[A(0)]…O4[T+1] in the As models. F, distance N6[A(0)]…O2P[A(+2)] in the As models. Moving average of a 50-snapshot window is shown in all panels. Figure S4. Occluded area (inaccessible to a 1.4 Å probe) between Phe111 side chain and A(+1) base. The colors of the traces are the same as in Fig. S1. The dashed line indicates the occluded area in the 1XC8 structure. Moving average of a 50-snapshot window is shown. Figure S5. A, Cumulative distribution of the occurrence of hydrogen bonds in the Lla-Fpg–DNA complex. B, Overall reproducibility of hydrogen bonds in replicate PRO-GLH runs. Dots show the coefficient of variation for the occurrence of a particular hydrogen bond calculated over four replicates plotted against the mean occurrence of the bond. The histograms show the distribution of the mean occurrence. The scale in all panels is the same. Numbers above the graphs indicate the percentage of hydrogen bonds with the mean occurrence >90%. F [file 12900_2017_75_MOESM1_ESM.pdf]
