# Supplementary material for: Molecular dynamics simulation of the opposite-base preference and interactions in the active site of formamidopyrimidine-DNA glycosylase
Source: BMC Struct Biol. 2017 May 8;17:5. doi: 10.1186/s12900-017-0075-y (PMC5422863; doi:10.1186/s12900-017-0075-y)
Supplement: Supplementary file 2 — pK a of Pro1 and Glu2 in selected Fpg structures. (DOC 42 kb) [file 12900_2017_75_MOESM2_ESM.doc]

**Additional file 2: Table S1.** p*K*_a_ of Pro1 and Glu2 in selected Fpg structures

| **PDB ID** | **p*K*_a_ Pro1** | **p*K*_a_ Glu2** | **Species** | **Type of structure** | **Reference** |
| --- | --- | --- | --- | --- | --- |
| Reference value*^a^* | 8.00 | 4.50 | – | – | [48] |
| Starting structure*^b^* | 6.00 | 6.72 | *Lla*-Fpg | complex with dsDNA (oxoG:C) | this work |
| 1XC8 | 5.01 | 7.62 | *Lla*-Fpg | complex with dsDNA (carba-fapyG:C) | [23] |
| 1EE8 | 7.15 | 6.40 | *Tth*-Fpg | free enzyme | [18] |
| 1PM5 | 6.36 | 8.14 | *Lla*-Fpg | complex with dsDNA (F:C)*^c^* | [24] |
| 1PJI | 7.65 | 8.05 | *Lla*-Fpg | complex with dsDNA (pAP site:C)*^d^* | [24] |
| 1L1T | 7.39 | 7.14 | *Bst*-Fpg | complex with dsDNA (rAP site:C)*^e^* | [20] |
| 1L2C | 7.35 | 7.43 | *Bst*-Fpg | complex with dsDNA (rAP site:T)*^e^* | [20] |
| 1L2D | 6.87 | 7.27 | *Bst*-Fpg | complex with dsDNA (rAP site:G)*^e^* | [20] |
| 2F5O | 6.95 | 6.22 | *Bst*-Fpg | cross-link with undamaged dsDNA*^f^* | [25] |
| 2F5P | 6.38 | 6.41 | *Bst*-Fpg | cross-link with undamaged dsDNA*^f^* | [25] |
| 2F5N | 7.05 | 6.04 | *Bst*-Fpg | cross-link with undamaged dsDNA*^f^* | [25] |

*^a^*p*K*_a_ of isolated terminal Pro (N1) and Glu (side chain carboxyl).

*^b^*1XC8 prepared as described in Methods.

*^c^*F, (3-hydroxytetrahydrofuran-2-yl)methyl phosphate (tethahydrofuran AP site), an uncleavable AP site analog.

*^d^*pAP, 3-hydroxypropyl phosphate (propanediol AP site), an uncleavable AP site analog.

*^e^*rAP, 2,3,5-trihydroxypentyl phosphate (reduced AP site), an uncleavable AP site analog.

*^f^*Structures 2F5O, 2F5P, and 2F5N contain undamaged DNA of different sequence.
